# The trend of self-harm incidence rates among adolescents in low- and lower-middle-income countries and its associated contextual factors

**DOI:** 10.1017/gmh.2026.10241

**Published:** 2026-06-02

**Authors:** Joshua Kirabo Sempungu, Minjae Choi, Hanul Park, Eun Hae Lee, Joon Hee Han, Sujeong Yu, Yo Han Lee

**Affiliations:** 1 Program in Public Health, Korea University Graduate School, Seoul, Republic of Korea; 2 Department of Preventive Medicine, Korea University College of Medicine, Seoul, Republic of Korea; 3 Institute for Future Public Health, Korea University, Seoul, Republic of Korea; 4 Program in Korean Unification Studies, Yonsei University, Seoul, Republic of Korea; 5 Graduate School of Social Welfare, Yonsei University, Seoul, Republic of Korea

**Keywords:** self-harm, suicide, adolescents, epidemiology, LMICs

## Abstract

This study examined trends in adolescent self-harm incidence rates and associated contextual factors among 77 low- and lower-middle-income countries. Annual sex-specific self-harm incidence rates for ages 10–19 years were obtained from the Global Burden of Disease 2021 database. Joinpoint regression assessed trends while country and year fixed-effects models examined their association with self-harm incidence rates. Median incidence rates declined from 35.9 to 35.3 per 100,000 among males and from 40.8 to 38.1 among females. Average annual percentage changes (AAPCs) among males were negative in 40 countries and positive in 37 (maximum 2.16%); among females, AAPCs were negative in 47 and positive in 30 (maximum 3.64%). Male self-harm incidence rates were positively associated with drug use exposure, adolescent fertility, new HIV infections, rule of law and unemployment, and negatively with control of corruption, alcohol use and urban population. Female incidence rates were positively associated with adolescent fertility rates, drug use, rule of law, mean years of schooling and labor force participation, and negatively with alcohol and tobacco use exposure, control of corruption, regulatory quality and sociodemographic index. These sex-specific differences in trends and contextual factors emphasize the need for integrated reproductive, mental and substance use targeted country-level prevention strategies.

## Impact statement

Adolescent self-harm research remains important in developing countries, where 73% of global suicides occur. However, to our knowledge, no previous study has examined trends in self-harm incidence in low- and lower-middle-income countries (LLMICs) or analyzed the national-level contextual factors associated with it. Using data from 77 LLMICs over 22 years, this study examines country-level trends in self-harm incidence rates among adolescents aged 10–19 and associated contextual factors. The findings reveal substantial heterogeneity, with incidence rates decreasing in 47 countries among females and 40 among males while increasing in 30 and 37 countries, respectively. Several contextual factors showed significant associations with self-harm incidence rates. For both sexes, the adolescent fertility rate, exposure to drug use and the rule of law were positively associated, while control of corruption was negatively associated. Sex-specific associations included new human immunodeficiency virus (HIV) infections, urban population and male unemployment rates and labor force participation rates, mean years of schooling and the sociodemographic index among females. These ecological associations in countries with high heterogeneity in self-harm incidence trends have important implications for global mental health policy and practice. They identify potential areas for population-level intervention research, including integrated reproductive and mental health services for adolescents, substance use prevention and governance strengthening. For public health systems, findings suggest potential value in exploring mental health integration in human immunodeficiency virus treatment and prevention programs for males and examining the mental health impacts of gender equity initiatives for employment and education of girls. Beyond identifying research priorities, the findings contribute to global mental health by providing the first comprehensive, cross-national analysis of contextual factors associated with adolescent self-harm in LLMICs. Although ecological studies cannot establish individual-level causation, population-level patterns could provide a basis for advocacy for mental health integration into sustainable development programs and highlight areas of further research.

## Introduction

According to the World Health Organization (WHO), among people of all ages, 720,000 individuals die by suicide worldwide each year, with 73% of these deaths occurring in developing countries (World Health Organization, 2024). Among young people aged 15–24 years, suicide is the third leading cause of death globally (World Health Organization, 2024). Self-harm is defined as the deliberate bodily damage inflicted on oneself, including both suicidal acts (self-harm with intent to die) and nonsuicidal self-injury (Hawton et al., 2012; 2015). Self-harm is a known risk factor for subsequent suicide, increasing risk by more than 30 times compared to the general population (Hawton et al., 2015). Understanding self-harm is therefore important for suicide prevention.

Self-harm often begins in adolescence, with self-injurious behavior emerging around age 13 (Gillies et al., 2018), and the incidence of self-harm is known to rise significantly during adolescence (Moran et al., 2024). Among adolescents (ages 10–19), UNICEF estimates that 46,000 die by suicide every year (United Nations Children’s Fund, 2021). Between 1990 and 2021, global suicide mortality among adolescents decreased by −1.6% annually (Yan et al., 2024). While global trends show a decline, significant regional heterogeneity exists, with Central and Tropical Latin America and Southern sub-Saharan Africa regions experiencing increases in suicide mortality (Yan et al., 2024). Southern Latin America has also been found to have the highest rate of increase in self-harm incidence previously (Tan et al., 2025). Many low- and lower-middle-income countries (LLMICs) are located in these regions (Metreau et al., 2024). In particular, a previous study showed that, at the global level, Southern sub-Saharan countries like Lesotho had the highest rate of increase for suicide mortality over 20 years among 10–24 year-olds, and low-middle sociodemographic index (SDI)-ranked countries had the highest suicide mortality rate until 2021 (Yan et al., 2024). This highlights the need for a better understanding of adolescent self-harm among LLMICs.

Population-level contextual factors, including national indicators such as demographic, economic, neighborhood, environmental and social and cultural events, are recognized as determinants of mental health. Lund et al., (2018)’s conceptual framework describes how these macro-level structural factors, including social stratification, economic policies, governance and others, shape intermediate determinants like living and working conditions and social support structures, which affect mental health outcomes through chronic stress, reduced resource access, social exclusion and trauma exposure (Lund et al., 2018). These factors, usually measured at the national level annually, have been termed contextual factors (Lange et al., 2023), macro-level factors (Andoh-Arthur and Adjorlolo, 2021) and nation-level factors (Rajkumar, 2023) in previous literature. These factors could be critical intervention points for population mental health outcomes, including self-harm.

Previous literature associated self-harm with socioeconomic factors (poverty, happiness, economic inequality, social capital; Bantjes et al., 2016; Lund et al., 2018; Lange et al., 2023; Rajkumar, 2023), health systems (health expenditure, health practitioners; Lange et al., 2023), substance use-related factors (alcohol use, drug use; Rizk et al., 2021; Athey et al., 2025) and governance (Zhang, 2022). Importantly, these associations differ by sex. Lange et al. (2023) found that among males, homicide, drug use and alcohol use were associated with suicide mortality, while among females, physician ratios and education inequality showed stronger associations, highlighting the importance of sex-specific examination (Lange et al., 2023).

Despite this recognition, research on contextual factors and adolescent self-harm in LLMICs remains limited. Of the eight relevant studies examining contextual factors and self-harm (Claveria, 2022; Er et al., 2023; Lange et al., 2023; Lari and Sefiddashti, 2023; Leveau, 2025; Lyu et al., 2025; Máté et al., 2025; Obama, 2025) that we reviewed, only 2 (Lari and Sefiddashti, 2023; Obama, 2025) focus on LLMICs. Additionally, among them, only Lange et al. (2023) examined sex-specific patterns in adolescents. This gap is concerning, given the unique challenges that LLMICs present, including distinct economic structures with large informal sectors (Lo Bue et al., 2022), limited mental health infrastructure (Lund et al., 2018) and stigma surrounding mental health (Aggarwal et al., 2017). These countries also lack adequate surveillance systems to capture self-harm episodes, limiting available data on self-harm (Moran et al., 2024). The differences suggest that contextual factors may operate differently in LLMICs than in high-income countries, necessitating further research.

This study addresses these gaps by examining sex-specific contextual factors associated with self-harm incidence rates in LLMICs. Analyzing data from 77 countries over 22 years (2000–2021), we hypothesized that socioeconomic development (gross domestic product [GDP] per capita and SDI), governance (control of corruption and rule of law), health system and demographic factors would show sex-specific associations with self-harm incidence rates based on previous literature (Andoh-Arthur and Adjorlolo, 2021; Lange et al., 2023; Rajkumar, 2023). Our objectives were to (1) establish the trend of self-harm incidence rates among adolescents aged 10–19 across 77 LLMICs and (2) examine sex-specific national-level contextual factors associated with changes in these rates. This research will inform self-harm and suicide prevention policy in LLMICs by identifying population-level intervention target areas.

Self-harm incidence rates derive from Global Burden of Disease (GBD) 2021 estimates, defining self-harm as deliberate bodily damage resulting in death or medically attended injury (ICD-9: E950-E959; ICD-10: X60-X64.9, X66-X84.9, Y87.0) (Ferrari et al., 2024; Global Burden of Disease 2021, 2024).

## Methods

### Study design and data

In this study, we utilized an ecological study design to examine the country-level contextual factors associated with sex-specific self-harm incidence in low- and lower-middle-income countries using the GBD 2021 (Ferrari et al., 2024). The ecological design used in this study was appropriate for generating hypotheses about population-level factors, but it cannot establish individual-level causality (Wakefield, 2009). GBD 2021 included estimates of incidence, prevalence, mortality and disability-adjusted life years, classified by location, age group and sex, for 371 causes across 204 countries and territories. The study utilized data from hospital records, emergency department records, insurance claims and population-representative surveys (Ferrari et al., 2024). Further descriptions of the methodology used by the GBD 2021 study have been published previously (Brauer et al., 2024; Ferrari et al., 2024). We obtained annual sex-specific self-harm incidence data for the ages 10–19 from the Institute for Health Metrics and Evaluation (IHME)’s GBD database for the years 2000 to 2021. We included only data from 77 countries classified by the World Bank as low-income and lower-middle-income in 2023. Low-income countries were 26 economies with a gross national income (GNI) of less than or equal to 1,135 United States Dollars (USD) in 2021. Lower-middle-income countries were 51 economies with a GNI between 1,136 USD and 4,495 USD (Metreau et al., 2024).

Contextual factors were selected based on existing theoretical frameworks of the social determinants of mental health (Lund et al., 2018) linking demographic, economic, neighborhood, environmental, and social and cultural events to population mental health. These domains are consistent with prior ecological studies examining self-harm and suicide (Kim et al., 2019; Er et al., 2023; Lange et al., 2023; Lari and Sefiddashti, 2023; Rajkumar, 2023; Leveau, 2025; Lyu et al., 2025; Obama, 2025). We obtained several contextual factors from the GBD database (Institute for Health Metrics and Evaluation (IHME), 2023), the World Bank databank (World Bank, 2024) and the United Nations Development Programme human development reports (UNDP, 2025). The contextual factors considered for inclusion included the summary exposure values (SEVs) for high alcohol use, childhood sexual abuse, drug abuse, intimate partner violence, high body mass index, sub-optimal temperature, tobacco use and unsafe water, sanitation and hand washing. They also included the adolescent fertility rate, urban population, primary completion rate, urban population growth rate, universal healthcare service coverage index, unemployment, physicians per 1,000 people, new HIV infections, youth HIV prevalence, health expenditure per capita, health expenditure as a percentage of the GDP, the human capital index, SDI, ratio of young literate females to males and a series of human development indicators including the labor force participation rate. Some contextual factors were sex-specific, as shown in Supplementary Materials (Table S1).

### Data analysis

We conducted a joinpoint regression analysis to estimate the trends in the incidence of self-harm among adolescents over the period between 2000 and 2021. The analysis was selected because it identifies statistically significant changes in trends without imposing a predetermined trajectory. This makes it more appropriate than linear regression for longitudinal data where trend shifts are expected (Kim et al., 2022). The joinpoint regression analysis fits linear segments into the data based on a set number of maximum joinpoints, determined by the number of data points (4 in this case), and applies a Monte Carlo permutation method to select the best model fit, choosing the model for which no additional segment would produce a statistically significant linear trend. Using joinpoint regression, the slope coefficient for each linear segment is estimated as the annual percentage change (APC), assumed to remain constant at the previous year’s rate. The Joinpoint Regression program presents the APC with confidence intervals (CIs), which are calculated parametrically. In this study, we used the average annual percentage change (AAPC), which is a summary measure estimated as a weighted average of the APCs from the joinpoint model. Further explanations about the methodology of joinpoint regression have been published previously (Kim et al., 2022). Joinpoint regression identifies points where statistically significant changes in trend occur, allowing for a more nuanced understanding than simple linear trends. The AAPC represents the overall trend across the entire period, accounting for any changes in trajectory. In this study, the accuracy of AAPC outputs was assessed using 95% CIs, and results with *p*-values >0.05 are indicated in the corresponding figures as nonsignificant AAPCs. The analysis was performed using the Joinpoint Regression Program 5.3.0 (National Cancer Institute, 2024), and the AAPCs of self-harm incidence rates in each country were visualized using R’s *“ggplot2”* package (Wickham, 2011).

For contextual factor analysis, the data acquired were assessed for missing values and prepared for imputation, since all countries had missing data on at least one variable, and 29 variables had missing data. Previous literature examining the extent to which imputation could be done found that 50% or more missingness warranted alterations and wide deviations from complete cases (Junaid et al., 2025). In the analysis before imputation, we excluded variables with more than 50% missingness in the Supplementary Materials (Table S2). We then utilized stratified median imputation using the geographic region, income level and year to impute missing values, after which Afghanistan was also excluded, as it remained with missing data (McDonald et al., 2015). Median imputation was used because of its robustness to outliers and preservation of the data’s central tendency, considering many extreme values are common in national-level indicators (McDonald et al., 2015). Countries with missing data after imputation, as shown in Supplementary Materials (Table S3), including Afghanistan, North Korea, Syria and Yemen, were excluded. All variables were then log-transformed to address skewness and assume normality.

We conducted a series of regression analyses to select contextual factors associated with self-harm incidence rates. First, univariate mixed-effects regression was used to investigate each factor associated with self-harm incidence rates (both log-transformed) with effects for year and country (Supplementary Materials [Tables S4, S5]). If a factor showed statistical evidence (*p*-value <0.05), it was retained for further analyses. Then, Pearson’s correlation coefficients were estimated among the remaining variables, and variables with high correlations (i.e., *r* = ±0.7 to ±1.0) were identified (Supplementary Materials [Tables S6 and S7]). This is consistent with existing literature on correlation thresholds for multicollinearity in regression modeling (Dormann et al., 2013). Among these, those with the largest effect sizes with lower *p*-values from the univariate mixed-effects analysis were kept. In cases where these had nearly identical effect sizes and *p*-values, the one with a lower variance inflation factor was retained (Supplementary Materials [Tables S6 and S7]). All the remaining variables were considered for inclusion in the final model. The final model was selected through stepwise backward elimination using the Akaike information criterion (AIC) for model comparison, removing variables sequentially until AIC no longer improved.

To assess model stability, we conducted bootstrap resampling (1,000 iterations) to examine coefficient stability; all variables showed minimal bias ranges (males = −0.01 to 0.01 and females = −0.01 to 0.05). This supported the robustness of our findings (Supplementary Materials [Tables S8]). However, among females, SEV drug use showed wider uncertainty (95% CI: −0.01, −0.64), suggesting its association with self-harm incidence rates should be interpreted with caution in the female model.

To select between the use of random and fixed effects, we employed the Hausman test (Hausman, 1978). The Hausman test compares the coefficient estimates from the fixed-effects and random-effects models. Under the null hypothesis, random effects are assumed to be consistent and efficient; however, if the test returns a significant result, it indicates that the individual-specific effects are correlated with the predictor variables, violating the random effects assumption and suggesting the fixed-effects model is more favorable (Hausman, 1978). In this study, the test returned significant results for both sexes (*X* ^2^ = 2,181.4, degrees of freedom (df) = 13, *p*-value <0.001 for females and *X* ^2^ = 154.65, df = 9, *p*-value <0.001 for males), indicating that country-specific effects were likely correlated with the macro-level factors considered in the final model. Thus, the final model included two-way fixed effects for both country and year. Variables that returned nonsignificant results (*p* > 0.05) were excluded in both the male and female models.

The final male model included variables: adolescent fertility rate, control of corruption, rule of law, SEV high alcohol use, SEV drug use, urban population, young people newly infected with HIV and youth unemployment rate. The final female model included variables: adolescent fertility rate, control of corruption, labor force participation rate, mean years of schooling, regulatory quality, rule of law, SDI, SEV high alcohol use, SEV drug use and SEV tobacco use. Correlations between self-harm incidence rates and the variables in the final selected model are included in Supplementary Materials (Figures S1 and S2).

Given that self-harm incidence rates and all other variables were log-transformed, coefficients represent elasticities, which are interpreted as the percent change in incidence rates associated with a 1% change in the predictor (Benoit, 2011; Wooldridge, 2016).

A sensitivity analysis was conducted to verify that multiple imputation did not introduce bias in the results, using the final model and complete-case analyses for countries and variables across the years. The results yielded did not differ significantly from those reported in the final model and are included in Supplementary Materials ([Tables S9]). We also conducted additional analyses using variables in the final models by region. The regional analysis showed heterogeneity, with some regions yielding estimates that differed from the combined analysis. The results are included in Supplementary Materials (Figure S3).

All analyses were carried out separately for females and males, significance set at *p*-value <0.05, and were carried out using R version 4.4.2 (R Core Team, 2024).

## Results

In 2000, median self-harm incidence was 35.88 per 100,000 (interquartile range [IQR]: 28.93–49.49) among males and 40.78 per 100,000 ([IQR]: 31.35–64.36) among females across the 77 countries. By 2021, this had changed to 35.25 ([IQR]: 28.48–46.1) and 38.12 ([IQR]: 29.8–67.1) among males and females, respectively. Substantial heterogeneity existed, with country-level rates in 2021 ranging from 12.73 to 183.43 among males and 22.2 to 200.02 among females.

### Trend analysis

**Figure 1.**
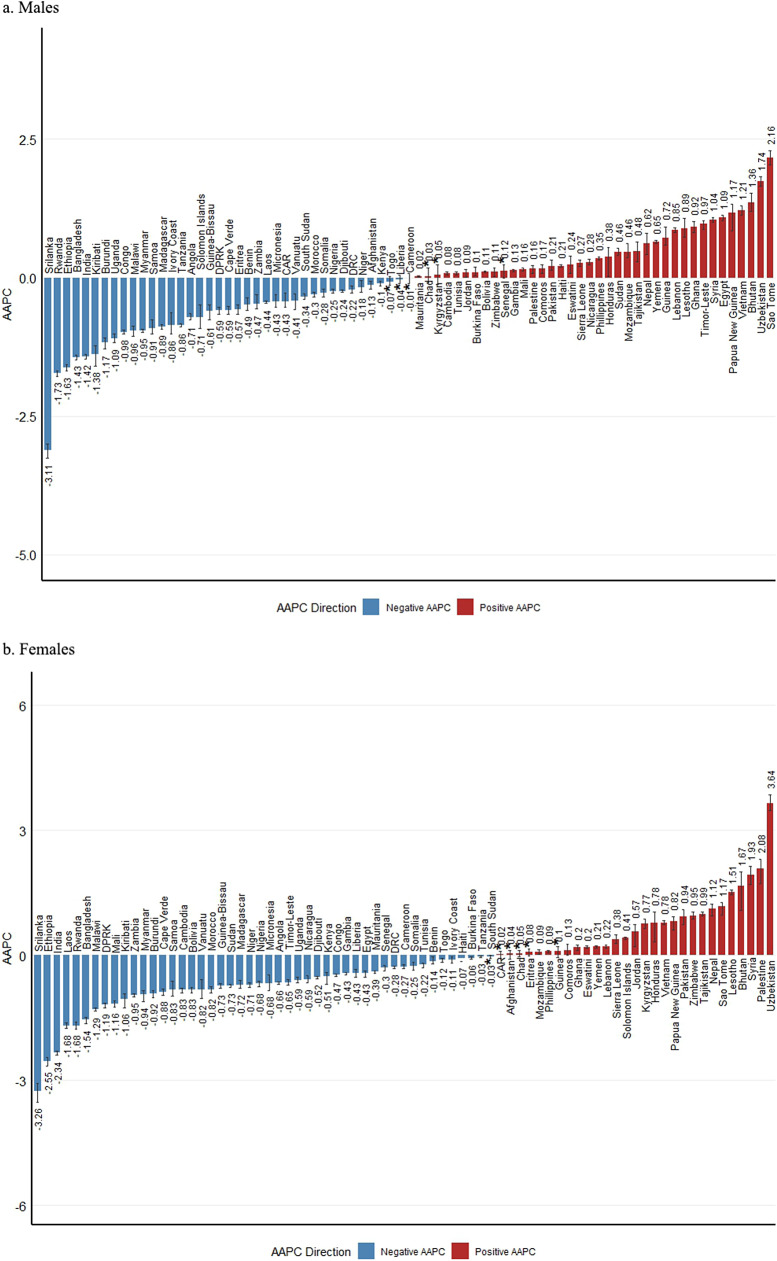
Self-harm incidence rate annual average percentage change for adolescents (ages 10–19) in 77 low- and lower-middle-income countries, 2000–2021. *Note*: AAPC, average annual percentage change. *Indicates *p* > 0.05. (a. male adolescents, b. female adolescents). Figure 1. long description.

The self-harm incidence rate per 100,000 adolescents aged 10–19 years in 77 low- and lower-middle-income countries is shown in Figure 1. Among male adolescents, 40 countries had decreasing AAPCs, among which Sri Lanka (−3.11% [95% CI: −3.25, −3.00]) had the highest percentage decrease, followed by Rwanda, Ethiopia, Bangladesh and India (Figure 1a) and among the 37 countries with increasing AAPCs, Sao Tome and Principe (2.16%) had the highest rate of increase, followed by Uzbekistan, Bhutan, Vietnam and Papua New Guinea.

Among females, 47 countries had reduced AAPCs, indicating gradual reductions in the overall incidence over the period, with Sri Lanka’s −3.26% (95% [CI]: −3.52, −3.07) being the lowest AAPC, followed by Ethiopia, India, Laos and Rwanda (Figure 1b). In contrast, 30 countries showed gradual overall increases, among which Uzbekistan’s 3.64% (95% CI: 3.48, 3.85) was the highest AAPC, followed by increases in Palestine, Syria, Bhutan and Lesotho.

Among the 13 countries that have declining trends among males and increasing trends in females, notable sex-specific differences were observed in Egypt (Male AAPC = 1.09 [95% CI: 1.03, 1.13], female AAPC = −0.43 [95% CI: −0.53, −0.37), Nicaragua (Male AAPC = 0.28 [95% CI: 0.23, 0.34], female AAPC = −0.59 [95% CI: −0.66, −0.49]), Sudan (Male AAPC = 0.46 [95% CI: 0.41, 0.53], female AAPC = −0.73 [95% CI: −0.77, −0.69]) and Timor Leste (Male AAPC = 0.97 [95% CI: 0.88, 1.03], female AAPC = −0.65 [95% CI: −0.71, −0.59]). Four countries had negative AAPCs for males and positive AAPCs for females. These were Afghanistan (Male AAPC = −0.13 [95% CI: −0.21, −0.06], female AAPC = 0.04 [95% CI: −0.09, 0.11]), Central African Republic, (Male AAPC = −0.43 [95% CI: −0.53, −0.28], female AAPC = 0.02 [95% CI: −0.06, 0.10]), Eritrea (Male AAPC = −0.57 [95% CI: −0.65, −0.49], female AAPC = 0.08 [95% CI: −0.01, 0.15]) and Solomon Islands (Male AAPC = −0.71 [95% CI: −0.91, −0.49], female AAPC = 0.41 [95% CI: 0.39, 0.43]).

There was substantial heterogeneity even among regions when the top 10 declines and increases for both sexes were examined. However, 6 of the 10 countries with the largest declines among males were in the WHO African region.

### Contextual factors for self-harm incidence

In Table 1, results showed that in the countries studied, while holding all other factors in the model constant, self-harm incidence rates among adolescent males increased with increases in adolescent fertility rates (estimate: 0.12; 95% CI: 0.09, 0.14), rule of law (estimate: 0.03; 95% CI: 0.02, 0.04), SEV for drug use (estimate: 0.23; 95% CI: 0.18, 0.29), young people newly infected with HIV (estimate: 0.03; 95% CI: 0.02, 0.03) and youth unemployment rate (estimate: 0.02; 95% CI: 0.01, 0.03).

**Table 1.** Fixed-effects regression model of associations between country-level contextual factors and self-harm incidence rates among male adolescents (ages 10–19) in 77 low- and lower-middle-income countries, 2000–2021 Table 1. long description.

Variable	Estimate	95% Confidence interval		*p*-value
		Lower	Upper	
Adolescent fertility rate	0.12	0.09	0.14	<0.001
Control of corruption	–0.03	−0.04	−0.02	<0.001
Rule of law	0.03	0.02	0.04	<0.001
SEV high alcohol use	−0.02	−0.03	0.00	0.011
SEV drug use	0.23	0.18	0.29	<0.001
Urban population	−0.05	−0.09	−0.01	0.006
Young people newly infected with HIV	0.03	0.02	0.03	<0.001
Youth unemployment rate	0.02	0.01	0.03	<0.001
R-squared = 0.19				
Adjusted R-squared = 0.13				

*Note:* Coefficients represent elasticities, which are interpreted as the percent change in incidence rates associated with a 1% change in the predictor.

*Abbreviation:* SEV, summary exposure value; HIV, human immunodeficiency virus.

On the other hand, self-harm incidence decreased with increases in control of corruption scores (estimate: −0.03; 95% CI: −0.04, −0.02), SEV for high alcohol use (estimate: −0.02; 95% CI: −0.03, 0.00) and urban population (estimate: −0.05; 95% CI: −0.09, −0.01).

In Table 2, results showed that in the countries studied, while holding all other factors in the model constant, self-harm incidence rates among female adolescents increased with increases in adolescent fertility (estimate: 0.19; 95% CI: 0.16, 0.22), labor force participation rates (estimate: 0.06; 95% CI: 0.04, 0.08), mean years of schooling (estimate: 0.03; 95% CI: 0.00, 0.05), rule of law (estimate: 0.03; 95% CI: 0.02, 0.04) and SEV for drug use (estimate: 0.13; 95% CI: 0.07, 0.19).

**Table 2.** Fixed-effects regression model of associations between country-level contextual factors and self-harm incidence rates among female adolescents (ages 10–19) in 77 low- and lower-middle-income countries, 2000–2021 Table 2. long description.

Variable	Estimate	95% CI (Lower)	95% CI (Upper)	*p*-value
Adolescent fertility rate	0.19	0.16	0.22	<0.001
Control of corruption	−0.02	−0.03	−0.01	<0.001
Labor force participation rate	0.06	0.04	0.08	<0.001
Mean years of schooling	0.03	0.00	0.05	0.04
Regulatory quality	−0.01	−0.03	0.00	0.007
Rule of law	0.03	0.02	0.04	<0.001
SDI	−0.41	−0.49	−0.33	<0.001
SEV high alcohol use	−0.06	−0.08	−0.04	<0.001
SEV drug use	0.13	0.07	0.19	<0.001
SEV tobacco use	−0.11	−0.16	−0.07	<0.001
*R*-squared = 0.26				
Adjusted *R*-squared = 0.21				

*Note:* Coefficients represent elasticities, which are interpreted as the percent change in incidence rates associated with a 1% change in the predictor.

*Abbreviation:* SEV, summary exposure value; SDI, sociodemographic index.

However, self-harm incidence rates decreased with increases in control of corruption (estimate: −0.02; 95% CI: −0.03, −0.01), regulatory quality (estimate: −0.01; 95% CI: −0.03, −0.00), SDI (estimate: −0.41; 95% CI: −0.49, −0.33), SEV for alcohol use (estimate: −0.06; 95% CI: −0.08, −0.04) and SEV for tobacco use (estimate: −0.11; 95% CI: −0.16, −0.07).

Several associations warrant careful interpretation. The rule of law showed positive associations, potentially reflecting better detection and reporting in well-governed countries rather than causal harm. Similarly, the negative association between alcohol use and female self-harm may reflect cultural factors, with countries having higher female alcohol use also having more gender-egalitarian norms that are protective for other reasons.

After accounting for contextual factors, significant year fixed effects remained (F-test, *p* < 0.001), indicating unmeasured temporal trends, such as changing diagnostic practices, evolving social media influence or global policy shifts, that affect self-harm incidence.

## Discussion

Our findings show that although most countries have reductions in the incidence of self-harm over the study period, many countries have experienced overall gradual increases in the incidence of self-harm, while others experienced reductions, consistent with previous studies. There also exist gender-specific differences, as more countries experienced a reduction in self-harm incidence among females when compared to males.

We found that countries including Uzbekistan, Syria, Palestine and Bhutan are experiencing high rates of increase in self-harm over the study period, an area needing additional research to understand countries with a relatively lower burden on an increasing trend. Another major finding was the high level of heterogeneity even among countries in similar regions. Apart from countries in the WHO Africa Region, representing 6 out of 10 of the countries with the highest rate of decline in self-harm incidence rates among male adolescents during the study period, increases among males and both decreases and increases among female adolescents did not return major region-specific differences.

Adolescent fertility rate, an indicator of gender equality and development, was positively associated with self-harm incidence rates across both sexes. Higher adolescent fertility rates could indicate higher rates of teenage pregnancy in these countries. Among pregnant young women, there is previous research indicating that conflict with parents, pregnancy-related anxiety, intimate partner violence and perceived poor health are associated with suicide attempts among adolescent girls (Li et al., 2021; Quarshie et al., 2025). Critically, this suggests that integrated reproductive health and mental health services for adolescents could yield suicide and self-harm prevention benefits. Programs providing educational continuity, economic support and mental health screening for pregnant or parenting adolescents warrant evaluation as self-harm prevention. Adolescent fatherhood has also been found to increase the risk of suicide attempt before (Walker et al., 2025), which could partially explain adolescent fertility rates’ associations with male self-harm rates in this study. However, this explanation is limited by the possibility that not all adolescent pregnancies are fathered by adolescent males. Further research could examine this relationship in more detail.

Among females, higher labor force participation rates and mean years of schooling, both indicators of gender inequality, were associated with increases in the rate of self-harm incidence. This contradicts previous findings that showed that higher gender equality was correlated with better outcomes for suicide (Lari and Sefiddashti, 2023; Bertuccio et al., 2024; Obama, 2025). While these indicators reflect development and gender equality, their positive associations may be due to a “thwarted ambitions” phenomenon in LLMICs. Higher education attainment is usually associated with limited opportunities, particularly for women (Knipe et al., 2019; Pharris et al., 2023). In the same way, labor force participation may expose young women to reward-to-effort imbalances in pay and work assignments, which worsen mental health (Li et al., 2017). However, these associations differ significantly across countries (Yoong et al., 2024). Therefore, further studies of these relationships in these countries are needed. The findings, however, may indicate a need not only to expand opportunities but also to ensure quality employment and address structural barriers that hinder women’s aspirations. Mental health policy and research could consider understanding how development indicators may contribute to psychological distress when social protections lag behind individual achievements.

Adolescent drug use and alcohol exposure have a profound association with adolescent suicidal behavior (Testa et al., 2019; Zhan et al., 2024). Drug use showed positive associations with self-harm incidence rates for both sexes, consistent with the literature that has established a link between substance use and suicide behavior through impulsivity, depression and anxiety pathways (Conner and Ilgen, 2011; Testa et al., 2019; Zhan et al., 2024). Exposure to high alcohol use, however, showed negative associations with self-harm incidence rates for both sexes. The counterintuitive findings for exposure to high alcohol use may reflect ecological fallacy. Countries with higher substance use may have more liberal social norms, better detection and reporting systems or other protective factors that outweigh individual-level risks. Individual-level associations between alcohol and suicide risk are well-established; the national-level pattern likely reflects unmeasured confounding rather than protective effects. These findings indicate the need for strengthening prevention of substance use and treatment among adolescents, particularly for drug use, which showed consistent positive associations.

We also found out that among males, the increase in urban population was associated with decreases in the incidence rates of self-harm at the national level. Urban population growth is associated with increased access to services (including health products) and higher screening (Runkle et al., 2023). This relationship, however, could be dependent on a lot of factors, as urban employment has previously been linked to many health risks and was found to have an association with self-harm (Catalano et al., 2011; Lange et al., 2023).

Additionally, unemployment, a usual result of economic decline at the national level, has previously been linked to many health risks (Catalano et al., 2011; Lange et al., 2023) and was found to have a positive association with self-harm. Through reduced incomes, loss of social identity and diminished prospects, unemployment may worsen mental health and create an environment in which adolescents are susceptible to self-harm, resulting in higher incidence rates.

Among males, the number of young people infected with HIV was also found to be associated with increases in self-harm incidence rates. HIV has previously been associated with adolescent suicide attempts, exacerbated by HIV stigma and drug abuse (Kreniske et al., 2022). HIV stigma is highly prevalent in many of the countries studied (Mendez‐Lopez et al., 2024), worsening mental health through high social exclusion, feelings of isolation and rejection, which contribute to the defeat that motivates suicide attempts. (O’Connor and Kirtley, 2018; Willis et al., 2018; Ashaba et al., 2019). Even as HIV infections decline globally, targeted mental health support for HIV positive adolescents remains critical in combating suicide attempts and other forms of self-harm.

SDI was associated with the country-level inequality in self-harm. Although varying, results have predominantly indicated that countries with lower social and economic development have a higher burden of self-harm and suicide (Yan et al., 2024; Tan et al., 2025). SDI encompasses income per capita, average educational attainment among adults and fertility rates among women aged 25 and younger, covering a broader range of development indicators than economic prosperity alone. This reinforces the importance of multidimensional sustainable development in LLMICs, creating an environment where fewer adolescents are exposed to the risk of self-harm.

Governance components, including the rule of law, regulatory quality and control of corruption, were associated with self-harm incidence in our study. A previous study of 24 countries found that suicide rates were lower in countries with lower levels of corruption (Yamamura et al., 2012). This association has previously been found to differ between females and males (Yamamura et al., 2012). Poor governance could contribute to worse living environments, accelerating inequality, poor service delivery and high unemployment levels, all of which are known contributors to poor mental health (Zhang, 2022), which is a known factor in self-harm incidence (Turecki and Brent, 2016). In this study, however, the inverse relationship between the rule of law and self-harm incidence rates among adolescents in LLMICs, both male and female, needs further examination in future research to understand how it worsens self-harm incidence rates among adolescents in LLMICs, both male and female. It could be interpreted that countries with stricter rule of law may have better vital registration systems, healthcare access and reporting mechanisms, leading to higher observed self-harm rates than true increases. However, a critical challenge in ecological studies is realized in this interpretation, as some of these critical implications cannot be proven (Wakefield, 2009).

This study represents the first comprehensive analysis of sex-specific contextual factors associated with adolescent self-harm trends in LLMICs, examining 77 countries over 22 years using internationally comparable data from the World Bank and United Nations databases.

However, several limitations warrant consideration. First, GBD estimates vary in certainty depending on data availability, with low-data countries relying heavily on statistical modeling and regional patterns (Voigt and King, 2017; Bhutta, 2025). GBD self-harm estimates include both fatal and nonfatal intentional self-injury events that resulted in healthcare contact or death. These estimates likely underestimate true incidence as many self-harm events, particularly in LLMICs, do not result in formal healthcare contact due to stigma, access barriers and cultural factors. Additionally, the proportion of self-harm events that are medically attended varies substantially across countries, potentially introducing systematic bias into cross-country comparisons. This study used GBD 2021, the most recent release available at the time of analysis. Updated estimates in subsequent GBD releases (e.g., GBD 2025) may lead to differences in point estimates; future work will update these analyses accordingly.

Second, the ecological study design precludes individual-level causal inference (Wakefield, 2009). National-level associations may reflect unmeasured confounding, reverse causality, or measurement artifacts rather than causal relationships. The positive association with the rule of law exemplifies this concern, which likely reflects better reporting rather than harmful effects.

Third, addressing missing data in this study was done by excluding countries and variables with large proportions of missing values (>50%) and using multiple imputations with neighboring countries within the same region and income classification. This may introduce bias. However, similar results were achieved from complete-case analysis. Many other contextual factors could have been included in this study but were not due to missing data; some of them may not be known to us. These may include suicide legislation and policy and its reach and implementation, national happiness levels, death rate due to homicide, absolute and relative education inequality, among others that have been used in previous studies (Lange et al., 2023; Rajkumar, 2023).

Fourth, the moderate R-squared values (19% for males, 26% for females) indicate that measured contextual factors explain limited variance in self-harm incidence. Substantial unexplained variance likely reflects unmeasured factors, including mental health service accessibility, cultural attitudes toward suicide and self-harm, restriction policies and within-country heterogeneity that national averages obscure. After accounting for measured contextual factors, significant year fixed effects remained (*p* < 0.001), indicating unmeasured temporal trends such as changing diagnostic practices, evolving social media influence, or global policy shifts.

Fifth, we excluded four conflict-affected countries (Afghanistan, North Korea, Syria and Yemen due to missing data, where self-harm patterns and determinants may differ substantially, limiting the generalizability of the results.

Sixth, some contextual factors, including youth HIV prevalence and new HIV infections, were only available for the age group 15–24. We retained them given the substantial overlap with the adolescent age range we studied and the lack of more granular age-disaggregated data for these countries. Readers should note this limitation when interpreting the associations involving these variables. Additionally, the article excludes psychological factors captured at the national level, which are known associated factors of self-harm.

Our results indicate that macro-level contextual factors are associated with adolescent self-harm in LLMICs, but data and research design limitations persist in this context. Many counterintuitive results required further research. However, the findings inform further research by providing testable hypotheses that could be validated in individual-level longitudinal studies in LLMICs to establish causal paths, which could inform national policy and other interventions in these countries. There is also a need to invest in harmonizable data systems in these countries, which could reduce reliance on estimates when investigating mental health issues at both the national and global levels.

## Conclusion

This research adds to the growing body of global mental health by examining the trend of adolescent self-harm in LLMICs and quantifying the association between national-level contextual factors. Findings indicate sex-differentiated prevention priorities: for male adolescents, addressing drug use, urban vulnerability, HIV-related mental health and youth unemployment; for female adolescents, ensuring quality employment, structural support for educated women, tobacco use prevention and economic growth. Across both sexes, integrating reproductive and mental health, improving governance and reducing socioeconomic inequality at the national level could help create safer environments for adolescents. We emphasize inclusive development that addresses gender and age-specific health effects of development and further research on suicidal behavior in LLMICs.

## Supporting information

10.1017/gmh.2026.10241.sm001 Sempungu et al. supplementary material Sempungu et al. supplementary material

## Data Availability

All data used in this study are publicly available at the IHME GBD Data Tool (https://vizhub.healthdata.org/gbd-results/), the World Bank DataBank (https://databank.worldbank.org/home.a) and the UNDP Data Center (https://hdr.undp.org/data-center).

## Supplementary material

The supplementary material for this article can be found at http://doi.org/10.1017/gmh.2026.10241.

## Long descriptions

### Figure 1. Long description

The figure consists of two vertical panels, labeled a for Males and b for Females.

Panel a, Males:

The y-axis represents A A P C, ranging from negative 5.0 to positive 2.5. The x-axis lists 77 countries. Blue bars extending downward indicate a negative A A P C, while red bars extending upward indicate a positive A A P C. The data shows a trend from Sri Lanka at negative 3.11 to Sao Tome at positive 2.16. Significant decreases are seen in Sri Lanka, Ethiopia, and Rwanda. Significant increases are seen in Sao Tome, Uzbekistan, and Bhutan. Values near zero include Mauritania at 0.02 and Chad at negative 0.03.

Panel b, Females:

The y-axis represents A A P C, ranging from negative 6 to positive 6. The x-axis lists the same 77 countries. The data shows a wider variance than the male cohort, trending from Sri Lanka at negative 3.26 to Uzbekistan at positive 3.64. Major negative changes are noted for Sri Lanka, Ethiopia, and India. Major positive changes are noted for Uzbekistan, Palestine, and Syria.

In both charts, an asterisk next to a country name indicates a p-value greater than 0.05, signifying that the change is not statistically significant. A legend at the bottom of each panel identifies blue as Negative A A P C and red as Positive A A P C.

Navigate back to Figure 1..

### Table 1. Long description

The table contains five columns: Variable, Estimate, 95 percent Confidence Interval Lower, 95 percent Confidence Interval Upper, and a final column for p-values.

* Adolescent fertility rate: Estimate 0.12, Lower 0.09, Upper 0.14, p-value less than 0.001.

* Control of corruption: Estimate negative 0.03, Lower negative 0.04, Upper negative 0.02, p-value less than 0.001.

* Rule of law: Estimate 0.03, Lower 0.02, Upper 0.04, p-value less than 0.001.

* S E V high alcohol use: Estimate negative 0.02, Lower negative 0.03, Upper 0.00, p-value 0.011.

* S E V drug use: Estimate 0.23, Lower 0.18, Upper 0.29, p-value less than 0.001.

* Urban population: Estimate negative 0.05, Lower negative 0.09, Upper negative 0.01, p-value 0.006.

* Young people newly infected with H I V: Estimate 0.03, Lower 0.02, Upper 0.03, p-value less than 0.001.

* Youth unemployment rate: Estimate 0.02, Lower 0.01, Upper 0.03, p-value less than 0.001.

Model fit statistics at the bottom indicate an R-squared of 0.19 and an Adjusted R-squared of 0.13. Note that S E V stands for summary exposure value and H I V stands for human immunodeficiency virus.

Navigate back to Table 1..

### Table 2. Long description

The table contains five columns: Variable, Estimate, 95 percent C I (Lower), 95 percent C I (Upper), and p-value.

* Adolescent fertility rate: Estimate 0.19, C I 0.16 to 0.22, p-value less than 0.001.

* Control of corruption: Estimate negative 0.02, C I negative 0.03 to negative 0.01, p-value less than 0.001.

* Labor force participation rate: Estimate 0.06, C I 0.04 to 0.08, p-value less than 0.001.

* Mean years of schooling: Estimate 0.03, C I 0.00 to 0.05, p-value 0.04.

* Regulatory quality: Estimate negative 0.01, C I negative 0.03 to 0.00, p-value 0.007.

* Rule of law: Estimate 0.03, C I 0.02 to 0.04, p-value less than 0.001.

* S D I: Estimate negative 0.41, C I negative 0.49 to negative 0.33, p-value less than 0.001.

* S E V high alcohol use: Estimate negative 0.06, C I negative 0.08 to negative 0.04, p-value less than 0.001.

* S E V drug use: Estimate 0.13, C I 0.07 to 0.19, p-value less than 0.001.

* S E V tobacco use: Estimate negative 0.11, C I negative 0.16 to negative 0.07, p-value less than 0.001.

Model fit statistics at the bottom indicate an R-squared of 0.26 and an Adjusted R-squared of 0.21.

Navigate back to Table 2..
