# Supplementary material for: The trend of self-harm incidence rates among adolescents in low- and lower-middle-income countries and its associated contextual factors
Source: Glob Ment Health (Camb). 2026 Jun 2;13:e142. doi: 10.1017/gmh.2026.10241 (PMC13373272; doi:10.1017/gmh.2026.10241)
Supplement: Sempungu et al. supplementary material [file S2054425126102416sup001.docx]

Supplementary Materials

# Supplementary Tables:

## Table S1: Covariates considered for inclusion in the study

| **Covariate** | **Source** | **Sex-specific** | **Previous References** |
| --- | --- | --- | --- |
| Self-harm Incidence | IHME | O | (Davis Weaver et al. 2025; Yan et al. 2024) |
| Socio-demographic index (SDI) | IHME | X | (Yan et al. 2024) |
| SEV* alcohol use | IHME | O | (Lange et al. 2023; Zhou et al. 2024) |
| SEV* childhood sexual abuse | IHME | O | (Klonsky and Moyer 2008; Lange et al. 2023) |
| SEV* drug abuse | IHME | O | (Lange et al. 2023; Zhou et al. 2024) |
| SEV* intimate partner violence | IHME | O | (Lange et al. 2023) |
| SEV* high BMI | IHME | O | (Batty et al. 2010) |
| SEV* sub-optimal temperature | IHME | O | (Zhou et al. 2024) |
| SEV* tobacco use | IHME | O | (Lange et al. 2023; Zhou et al. 2024) |
| SEV* unsafe water, sanitation and handwashing | IHME | O | (Lange et al. 2023) |
| Adolescent fertility rate (per 1000 15-19) | World Bank | X | (Obama 2025) |
| Urban population | World Bank | X | (Lari and Sefiddashti 2023) |
| Primary completion | World Bank | O | (Lari and Sefiddashti 2023) |
| Urban population growth rate | World Bank | X | (Runkle et al. 2023) |
| UHC service coverage index | World Bank | X | (Ye et al. 2025) |
| Unemployment | World Bank | X | (Claveria 2022; Er et al. 2023; Lange et al. 2023; Lyu et al. 2025) |
| Physicians per 1000 people | World Bank | X | (Lange et al. 2023) |
| New HIV infections per year | World Bank | X | (Obama 2025) |
| Youth HIV prevalence (15-24) | World Bank | X | (Obama 2025) |
| Health expenditure per capita | World Bank | X | (Lange et al. 2023) |
| Health expenditure as % of GDP | World Bank | X | (Lange et al. 2023) |
| Human capital index | World Bank | X | (Rajkumar 2023) |
| Ratio of young literate females to males | World Bank | X | (Lari and Sefiddashti 2023) |
| GDP per capita | World Bank | X | (Er et al. 2023; Lyu et al. 2025) |
| Rule of Law | World Bank | X | (Obama 2025) |
| Voice and Accountability | World Bank | X | (Obama 2025) |
| Political Stability and Absence of Violence/Terrorism | World Bank | X | (Obama 2025) |
| Government Effectiveness | World Bank | X | (Obama 2025) |
| Regulatory Quality | World Bank | X | (Obama 2025) |
| Rule of Law | World Bank | X | (Obama 2025) |
| Control of Corruption | World Bank | X | (Obama 2025) |
| Labor force participation rate | UNDP | O | (Lari and Sefiddashti 2023; Obama 2025) |
| Mean years of Schooling | UNDP | O | (Lange et al. 2023; Lari and Sefiddashti 2023; Obama 2025) |
| Population Density | UNDP | X | (Lange et al. 2023; Rajkumar 2023) |
| Gender development index (GDI) | UNDP | X | (Rajkumar 2023) |
| Gender Inequality Index (GII) | UNDP | X | (Obama 2025) |
| Gross national income (GNI) | UNDP | O | (Lyu et al. 2025) |
| Human development index (HDI) | UNDP | O | (Rajkumar 2023) |

**Summary exposure value (SEV) - A measure (ranging from 0 to 100) of a population’s exposure to a risk factor that considers the extent of exposure by risk level and the severity of that risk’s contribution to disease burden.*

*BMI - Body mass index, UHC – Universal health coverage, HIV – Human immunodeficiency virus, GDP – Gross domestic product.*

*Sex-specific variables were downloaded from databases with separate values for females and males.*

## Table S2. Variable missingness between males and females

| Variable | Observations missing (%) | |
| --- | --- | --- |
|  | Males | Females |
| Human Capital Index | 87 | 87 |
| Literacy ratio | 78 | 78 |
| Universal Health Coverage Index | 69 | 69 |
| Physicians per 1000 population | 56 | 56 |

## Table S3: Countries excluded after imputation and their missing proportions

| Country | GDP per capita | Primary completion | New HIV Infections | HIV Prevalence | Health expenditure per capita | Health expenditure as % of GDP | Gross National Income | Labor force participation rate | Mean years of schooling | HDI | GDI | GII |
| --- | --- | --- | --- | --- | --- | --- | --- | --- | --- | --- | --- | --- |
| Afghanistan | 9 | 82 | 0 | 0 | 9 | 9 | 36 | 36 | 0 | 36 | 36 | 36 |
| DPRK | 100 | 96 | 100 | 100 | 100 | 100 | 100 | 100 | 100 | 100 | 100 | 100 |
| Syria | 0 | 23 | 0 | 0 | 27 | 27 | 0 | 0 | 0 | 0 | 0 | 0 |
| Yemen | 0 | 23 | 0 | 0 | 27 | 27 | 0 | 0 | 0 | 0 | 0 | 0 |

## Table S4: Univariate Analyses for Contextual Factors Associations with Self-harm Incidence (Males, log-transformed)

| Predictor | Estimate | SE | T-statistic | P-value | VIFs |
| --- | --- | --- | --- | --- | --- |
| SEV drug abuse | 0.25 | 0.03 | 9.02 | <0.001 | 3.83 |
| Adolescent fertility rate | 0.10 | 0.01 | 9.50 | <0.001 | 4.18 |
| New HIV cases | 0.03 | 0.00 | 7.49 | <0.001 | 4.69 |
| Youth HIV Prevalence | 0.04 | 0.01 | 6.78 | <0.001 | 6.92 |
| SEV WASH | -0.12 | 0.02 | -6.55 | <0.001 | 6.37 |
| Rule of law | 0.03 | 0.01 | 6.29 | <0.001 | 1.80 |
| Gross national income | -0.04 | 0.01 | -6.06 | <0.001 | 9.21 |
| Human development index | -0.11 | 0.02 | -5.88 | <0.001 | 11.79 |
| Socio-demographic index | -0.08 | 0.01 | -5.15 | <0.001 | 4.86 |
| Urban population | -0.03 | 0.01 | -4.66 | <0.001 | 2.74 |
| Alcohol per capita | -0.02 | 0.01 | -4.47 | <0.001 | 3.31 |
| Control of corruption | -0.02 | 0.01 | -4.27 | <0.001 | 1.47 |
| GDP per capita | -0.02 | 0.00 | -4.14 | <0.001 | 8.01 |
| SEV alcohol use | -0.03 | 0.01 | -4.00 | <0.001 | 4.02 |
| SEV tobacco | 0.08 | 0.02 | 3.76 | <0.001 | 2.20 |
| Population density | -0.04 | 0.01 | -3.54 | <0.001 | 1.90 |
| Labor force participation rate | 0.04 | 0.01 | 2.96 | 0.003 | 1.43 |
| Mean years of schooling | -0.03 | 0.01 | -2.82 | 0.005 | 3.73 |
| Health expenditure per capita | -0.01 | 0.00 | -2.81 | 0.005 | 8.53 |
| SEV high BMI | -0.03 | 0.01 | -2.81 | 0.005 | 3.09 |
| Youth unemployment | 0.01 | 0.01 | 2.51 | 0.012 | 1.55 |
| Gender development index | -0.12 | 0.05 | -2.46 | 0.014 | 2.56 |
| Gender inequality index | 0.04 | 0.02 | 2.15 | 0.032 | 3.65 |
| SEV Suboptimal temperature | 0.03 | 0.02 | 1.66 | 0.098 |  |
| Voice and accountability | 0.01 | 0.00 | 1.40 | 0.162 |  |
| SEV childhood sexual abuse | -0.02 | 0.02 | -1.14 | 0.256 |  |
| Regulatory authority | -0.01 | 0.01 | -1.06 | 0.290 |  |
| Urban population growth | 0.00 | 0.00 | -0.99 | 0.323 |  |
| Political stability and absence of war | 0.00 | 0.00 | 0.53 | 0.595 |  |
| Health expenditure as % of GDP | 0.00 | 0.01 | 0.39 | 0.695 |  |
| Government efficiency | 0.00 | 0.01 | 0.09 | 0.930 |  |

**Summary exposure value (SEV) - A measure (ranging from 0 to 100) of a population’s exposure to a risk factor that considers the extent of exposure by risk level and the severity of that risk’s contribution to disease burden.*

*BMI - Body mass index, UHC – Universal health coverage, HIV – Human immunodeficiency virus, GDP – Gross domestic product.*

## Table S5: Univariate Analyses for Contextual Factors Associations with Self-harm Incidence (Females, log-transformed)

| Predictor | Estimate | SE | T-statistic | p-Value | VIFs |
| --- | --- | --- | --- | --- | --- |
| Socio-demographic index | -0.24 | 0.02 | -14.24 | <0.001 | 5.05 |
| Adolescent fertility rate | 0.21 | 0.01 | 17.53 | <0.001 | 4.82 |
| Urban population | -0.09 | 0.01 | -11.07 | <0.001 | 4.20 |
| GDP per capita | -0.05 | 0.00 | -10.66 | <0.001 | 9.16 |
| HDI | -0.18 | 0.02 | -10.59 | <0.001 | 13.95 |
| Population density | -0.12 | 0.01 | -9.67 | <0.001 | 2.10 |
| SEV high BMI | -0.13 | 0.01 | -9.19 | <0.001 | 3.99 |
| Health expenditure per capita | -0.03 | 0.00 | -8.92 | <0.001 | 8.77 |
| Mean years of schooling | -0.08 | 0.01 | -8.56 | <0.001 | 4.23 |
| Alcohol per capita | -0.05 | 0.01 | -7.22 | <0.001 | 2.41 |
| SEV childhood sexual abuse | -0.17 | 0.02 | -7.57 | <0.001 | 1.89 |
| Labor force participation | 0.07 | 0.01 | 6.81 | <0.001 | 4.13 |
| SEV alcohol use | -0.07 | 0.01 | -5.73 | <0.001 | 2.84 |
| Youth HIV prevalence (15-24) | 0.04 | 0.01 | 5.50 | <0.001 | 6.24 |
| Gender inequality index | 0.13 | 0.02 | 6.04 | <0.001 | 3.78 |
| New HIV infections per year | 0.03 | 0.01 | 5.07 | <0.001 | 5.24 |
| Gender development index | -0.28 | 0.06 | -4.39 | <0.001 | 4.89 |
| Rule of law | 0.03 | 0.01 | 4.18 | <0.001 | 1.84 |
| GNI | -0.03 | 0.01 | -3.45 | 0.001 | 6.16 |
| SEV drug abuse | 0.11 | 0.04 | 3.25 | 0.001 | 3.40 |
| Control of corruption | -0.02 | 0.01 | -2.88 | 0.004 | 1.51 |
| SEV tobacco | 0.07 | 0.02 | 2.76 | 0.006 | 2.40 |
| SEV WASH | -0.05 | 0.02 | -2.25 | 0.025 | 5.81 |
| SEV suboptimal temperature | 0.04 | 0.02 | 2.22 | 0.027 | 2.68 |
| Regulatory authority | -0.01 | 0.01 | -2.08 | 0.038 | 1.02 |
| Youth unemployment | 0.01 | 0.01 | 1.73 | 0.085 |  |
| SEV IPV | -0.05 | 0.03 | -1.55 | 0.121 |  |
| Voice and accountability | 0.01 | 0.01 | 1.55 | 0.121 |  |
| Health expenditure as % of GDP | 0.01 | 0.01 | 1.22 | 0.223 |  |
| Urban population growth rate | 0.00 | 0.00 | -0.48 | 0.634 |  |
| Government efficiency | 0.00 | 0.01 | 0.43 | 0.666 |  |
| Political stability and absence of war | 0.00 | 0.00 | 0.22 | 0.825 |  |

*Summary exposure value (SEV) - A measure (ranging from 0 to 100) of a population’s exposure to a risk factor that considers the extent of exposure by risk level and the severity of that risk’s contribution to disease burden.*

*BMI - Body mass index, HIV – Human immunodeficiency virus, GDP – Gross domestic product, HDI- Human development index, GNI- Gross national income, SDI- socio-demographic index, WASH-Water, sanitation and hygiene, IPV-Intimate partner violence.*

## Table S6: Pearsson correlation for redundant covariates (Males)

| Highly Correlated Pairs | | |
| --- | --- | --- |
| Var 1 | Var 2 | Correlation Value |
| Health Expenditure per capita | GDP per Capita | 0.89 |
| HDI | GNI | 0.86 |
| SDI | HDI | 0.80 |
| Youth HIV Prevalence | New HIV cases | 0.79 |
| GDP per capita | GNI | 0.79 |
| Mean Years of Schooling | HDI | 0.75 |
| SEV Alcohol Use | Alcohol consumption per capita | 0.75 |
| GDP per capita | HDI | 0.74 |
| SDI | GNI | 0.73 |
| Health Expenditure per capita | HDI | 0.73 |
| Mean Years of Schooling | SDI | 0.72 |
| Health Expenditure per capita | GNI | 0.71 |
| SEV WASH | Adolescent Fertility rate | 0.70 |
| GII | Adolescent Fertility rate | 0.70 |
| GNI | SEV WASH | -0.74 |
| HDI | SEV WASH | -0.75 |
| SEV WASH | SEV Drug Use | -0.80 |

*Summary exposure value (SEV) - A measure (ranging from 0 to 100) of a population’s exposure to a risk factor that considers the extent of exposure by risk level and the severity of that risk’s contribution to disease burden.*

*BMI - Body mass index, HIV – Human immunodeficiency virus, GDP – Gross domestic product, HDI- Human development index, GNI- Gross national income, SDI- socio-demographic index, WASH-Water, sanitation and hygiene.*

## Table S7: Pearsson correlation for redundant covariates (Females)

| Highly Correlated Pairs | | |
| --- | --- | --- |
| Var 1 | Var 2 | Correlation Value |
| Health Expenditure per capita | GDP per Capita | 0.89 |
| GNI | HDI | 0.82 |
| HDI | SDI | 0.80 |
| Mean Years of Schooling | HDI | 0.80 |
| New HIV cases | Youth HIV Prevalence | 0.79 |
| GNI | GDP per Capita | 0.77 |
| Mean Years of Schooling | SDI | 0.74 |
| Health Expenditure per capita | HDI | 0.73 |
| HDI | GDP per Capita | 0.71 |
| SEV WASH | Adolescent Fertility rate | 0.70 |
| GII | Adolescent Fertility rate | 0.70 |
| GNI | SDI | 0.70 |
| SEV WASH | HDI | -0.71 |
| SEV WASH | SEV Drug Use | -0.72 |

**Summary exposure value (SEV) - A measure (ranging from 0 to 100) of a population’s exposure to a risk factor that considers the extent of exposure by risk level and the severity of that risk’s contribution to disease burden.*

*BMI - Body mass index, HIV – Human immunodeficiency virus, GDP – Gross domestic product, HDI- Human development index, GNI- Gross national income, SDI- socio-demographic index, WASH-Water, sanitation and hygiene.*

## Table S8: Bootstrap resampling (1000 iterations) to examine coefficient stability of the final model (males)

| **Variable** | **Original Estimate** | **Bootstrap Mean** | **Bootstrap SE** | **Bootstrap 95% CI** | | **Bias** |
| --- | --- | --- | --- | --- | --- | --- |
|  |  |  |  | **Lower** | **Upper** |  |
| **Males** | | | | | | |
| Adolescent fertility rate | 0.1169 | 0.1058 | 0.0623 | -0.041 | 0.208 | -0.0111 |
| SEV high alcohol use | -0.0167 | -0.0168 | 0.0243 | -0.0732 | 0.026 | -1.00E-04 |
| SEV drug use | 0.2325 | 0.2408 | 0.1282 | 0.011 | 0.5253 | 0.0083 |
| Control of corruption | -0.0279 | -0.0281 | 0.013 | -0.0538 | -0.002 | -2.00E-04 |
| Rule of law | 0.0335 | 0.0328 | 0.0169 | 0.0055 | 0.0726 | -7.00E-04 |
| Young people newly infected with HIV | 0.0263 | 0.0254 | 0.0141 | -0.0028 | 0.054 | -0.001 |
| Youth unemployment rate | 0.0197 | 0.0169 | 0.0158 | -0.0147 | 0.0474 | -0.0028 |
| Urban population | -0.0507 | -0.061 | 0.0813 | -0.2185 | 0.0932 | -0.0104 |
| Females | | | | | | |
| Variable | Original Estimate | Bootstrap Mean | Bootstrap SE | Lower | Upper | Bias |
| Adolescent fertility rate | 0.1914 | 0.1775 | 0.0713 | 0.004 | 0.3026 | -0.0139 |
| SEV high alcohol use | -0.0633 | -0.0648 | 0.0388 | -0.1464 | 0.0035 | -0.0015 |
| SEV drug use | 0.1291 | 0.1784 | 0.1621 | -0.0058 | 0.641 | 0.0493 |
| Control of corruption | -0.0194 | -0.0198 | 0.0113 | -0.042 | 0.0039 | -0.0004 |
| Regulatory Quality | -0.0145 | -0.0123 | 0.0096 | -0.0292 | 0.0076 | 0.0022 |
| Rule of law | 0.0317 | 0.0313 | 0.019 | 0.002 | 0.0736 | -0.0004 |
| Labor force participation rate | 0.0601 | 0.0565 | 0.0332 | -0.0068 | 0.1208 | -0.0036 |
| Mean years of schooling | 0.0263 | 0.0251 | 0.0334 | -0.0415 | 0.0922 | -0.0012 |
| Sociodemographic Index | -0.4124 | -0.4218 | 0.1445 | -0.6868 | -0.1328 | -0.0094 |
| SEV tobacco use | -0.1122 | -0.115 | 0.0794 | -0.2733 | 0.0437 | -0.0028 |

## Table S9: Complete case analysis using two-way fixed effects

| **Variable** | **Estimate (95%CI)** | **p-value** |
| --- | --- | --- |
| **Males** | | |
| Adolescent fertility rate | 0.1 (0.07 - 0.07) | <0.001 |
| SEV alcohol use | -0.01 (-0.03 - -0.03) | 0.06 |
| SEV drug use | 0.46 (0.4 - 0.4) | <0.001 |
| GDP per capita | -0.01 (-0.03 - -0.03) | 0.09 |
| Control of corruption | -0.03 (-0.04 - -0.04) | <0.001 |
| Rule of law | 0.07 (0.06 - 0.06) | <0.001 |
| Young people newly infected with HIV | 0.03 (0.02 - 0.02) | <0.001 |
| Youth unemployment rate | 0.02 (0.01 - 0.01) | <0.001 |
| Urban population | -0.08 (-0.12 - -0.12) | <0.001 |
| *R-squared = 0.28, Adjusted R-squared = 0.23, Unbalanced panel n = 62, T=11-22* | | |
| **Females** | | |
| Adolescent fertility rate | 0.24 (0.21 - 0.21) | <0.001 |
| SEV alcohol use | -0.04 (-0.07 - -0.07) | 0.004 |
| Alcohol consumption per capita | -0.01 (-0.03 - -0.03) | 0.11 |
| SEV childhood sexual abuse | 0.04 (-0.03 - -0.03) | 0.23 |
| SEV drug use | 0.14 (0.07 - 0.07) | <0.001 |
| Control of corruption | -0.02 (-0.04 - -0.04) | <0.001 |
| Regulatory quality | 0 (-0.02 - -0.02) | 0.69 |
| Rule of law | 0.03 (0.01 - 0.01) | <0.001 |
| Labor force participation rate | 0.01 (-0.01 - -0.01) | 0.24 |
| Mean years of schooling | 0.08 (0.05 - 0.05) | <0.001 |
| SDI | -0.56 (-0.66 - -0.66) | <0.001 |
| SEV Sub-optimal temperature | 0.01 (-0.03 - -0.03) | 0.50 |
| SEV Tobacco use | -0.14 (-0.19 - -0.19) | <0.001 |
| *R-squared = 0.32, Adjusted R-squared = 0.26, Unbalanced panel n = 70, T=3-22* | | |

# Supplementary Figures

## Figure S1: Forest plots indicating correlations between variables in the final model and self-harm incidence rates among female adolescents.

1. Adolescent Fertility Rate
2.
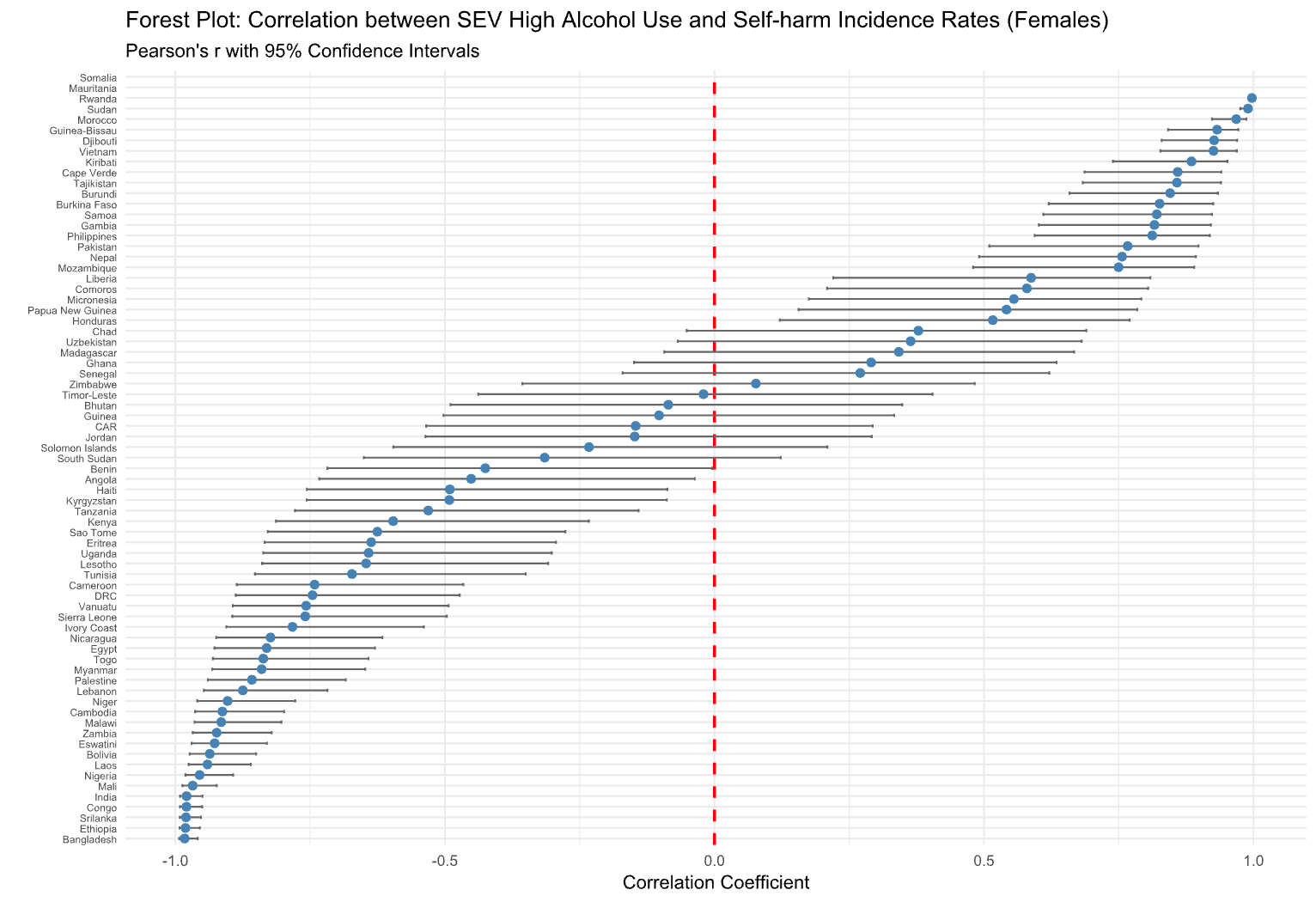

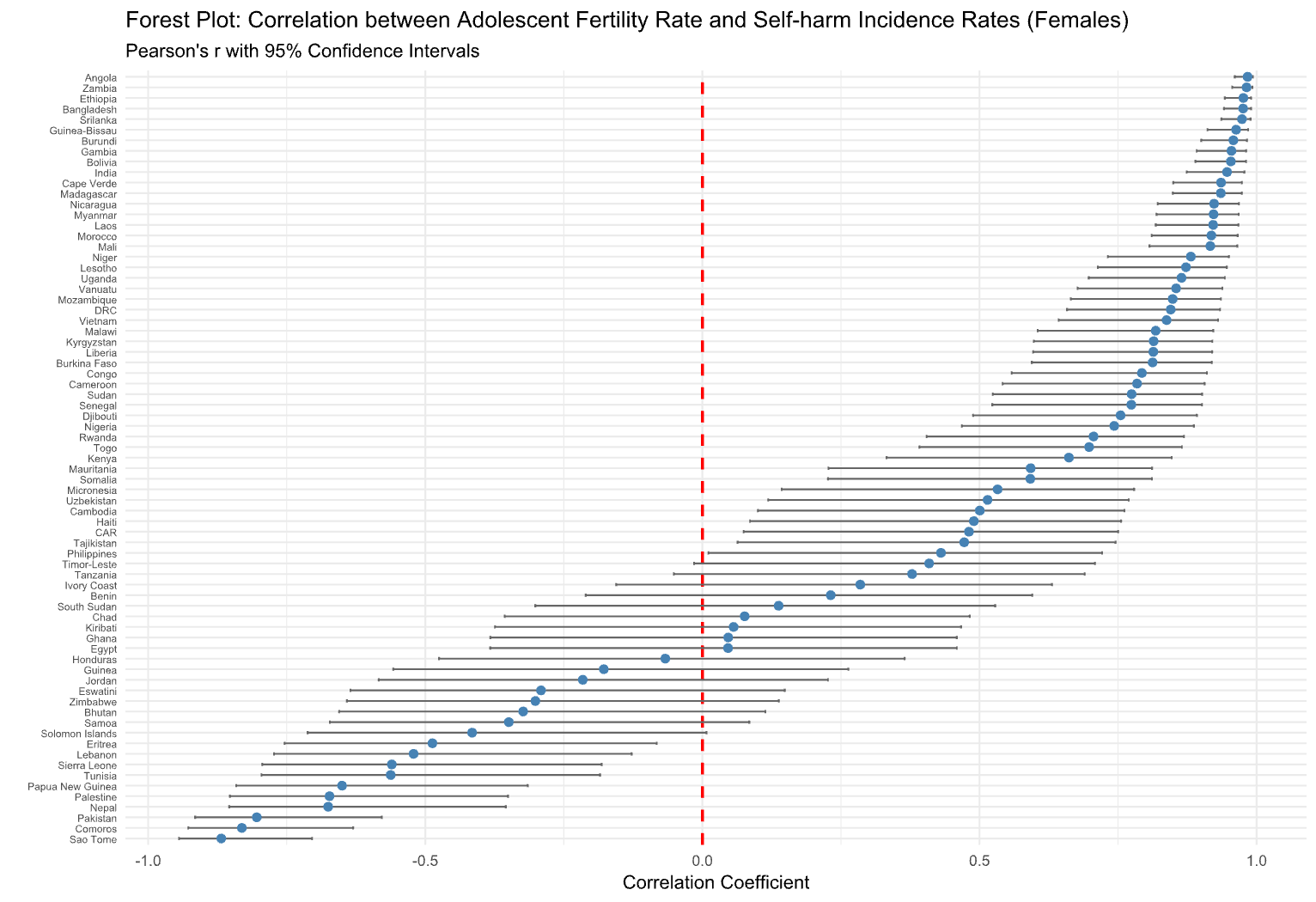
SEV High Alcohol Use
3.
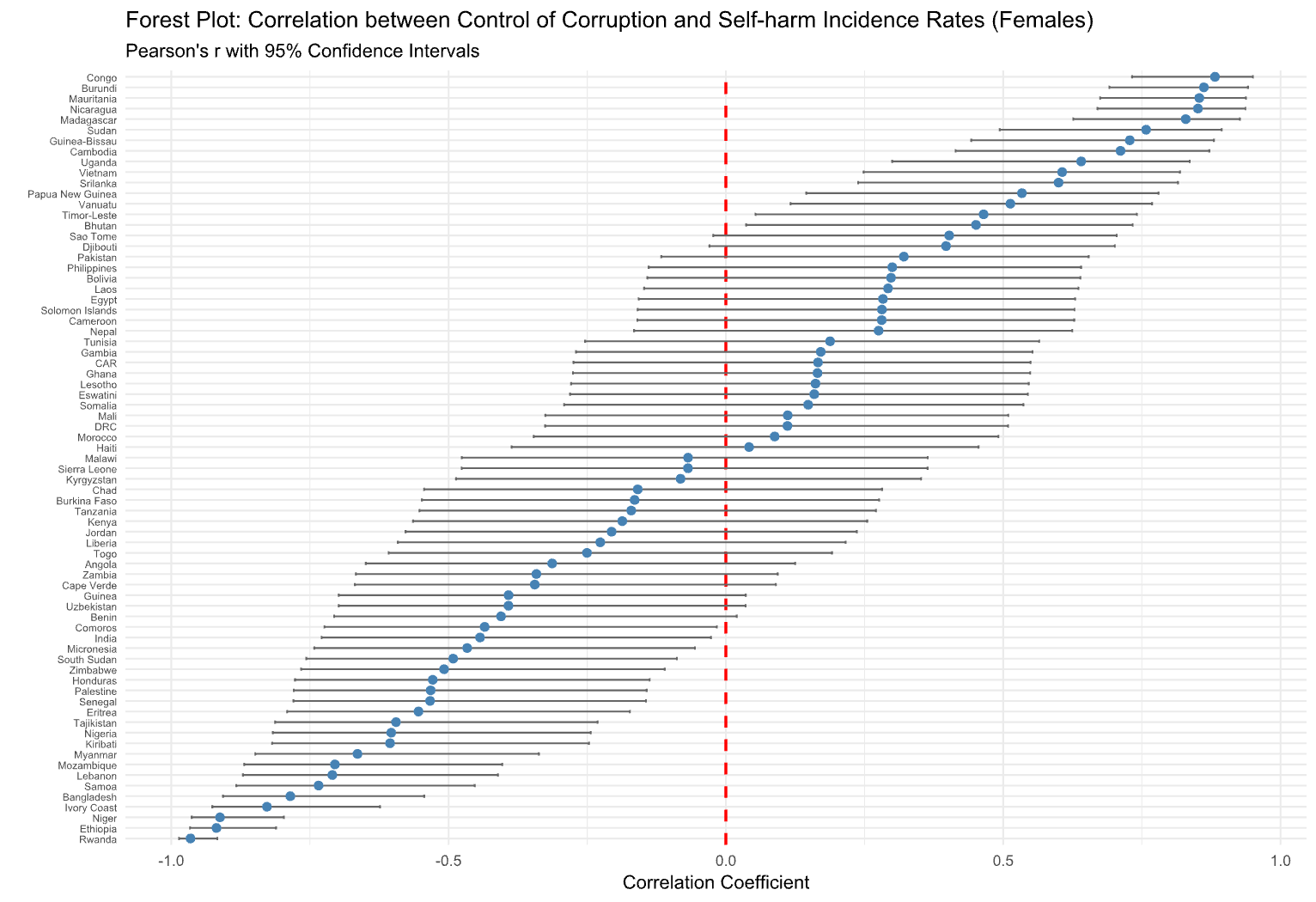
Control of Corruption
4.
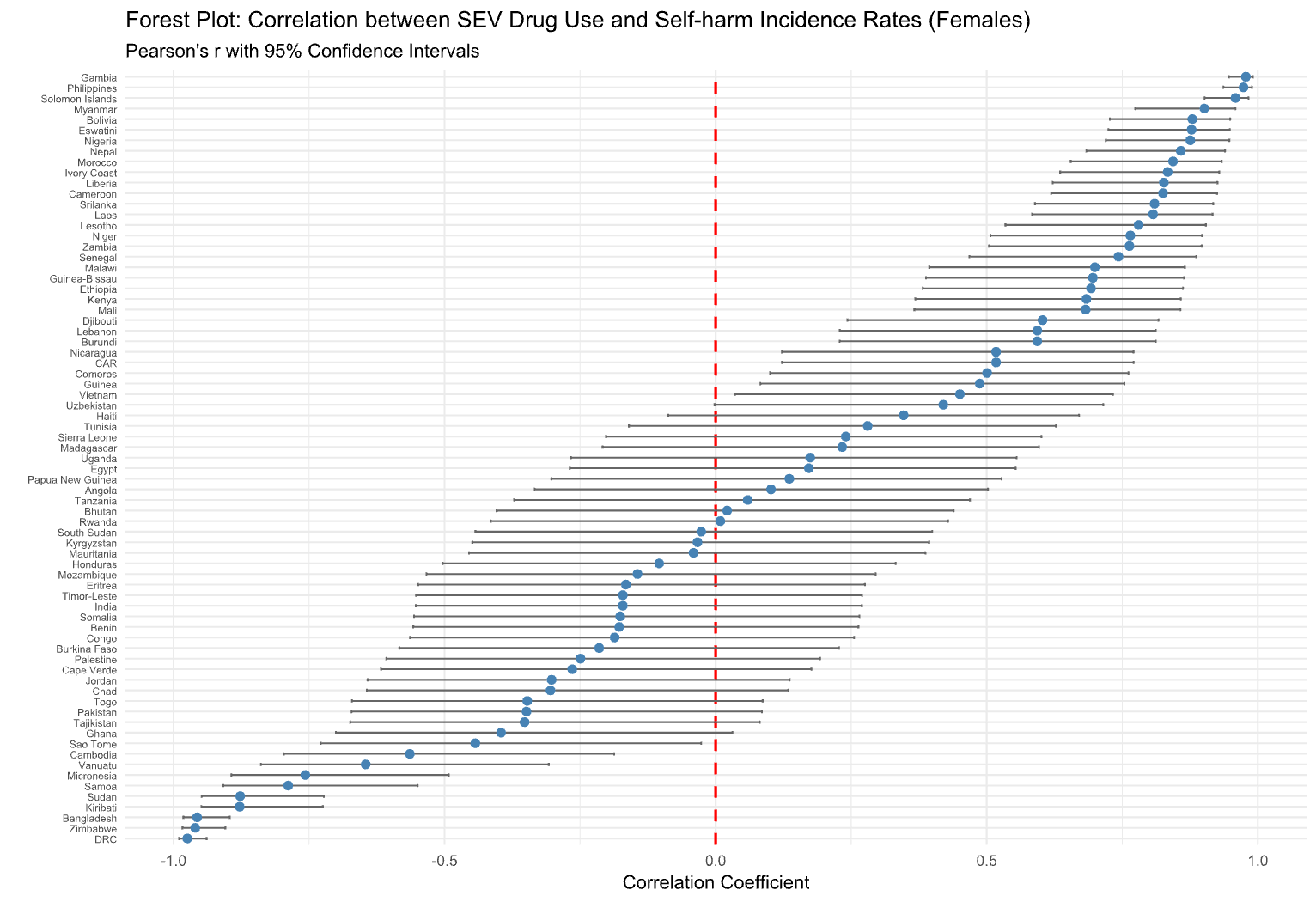
SEV Drug Use
5.
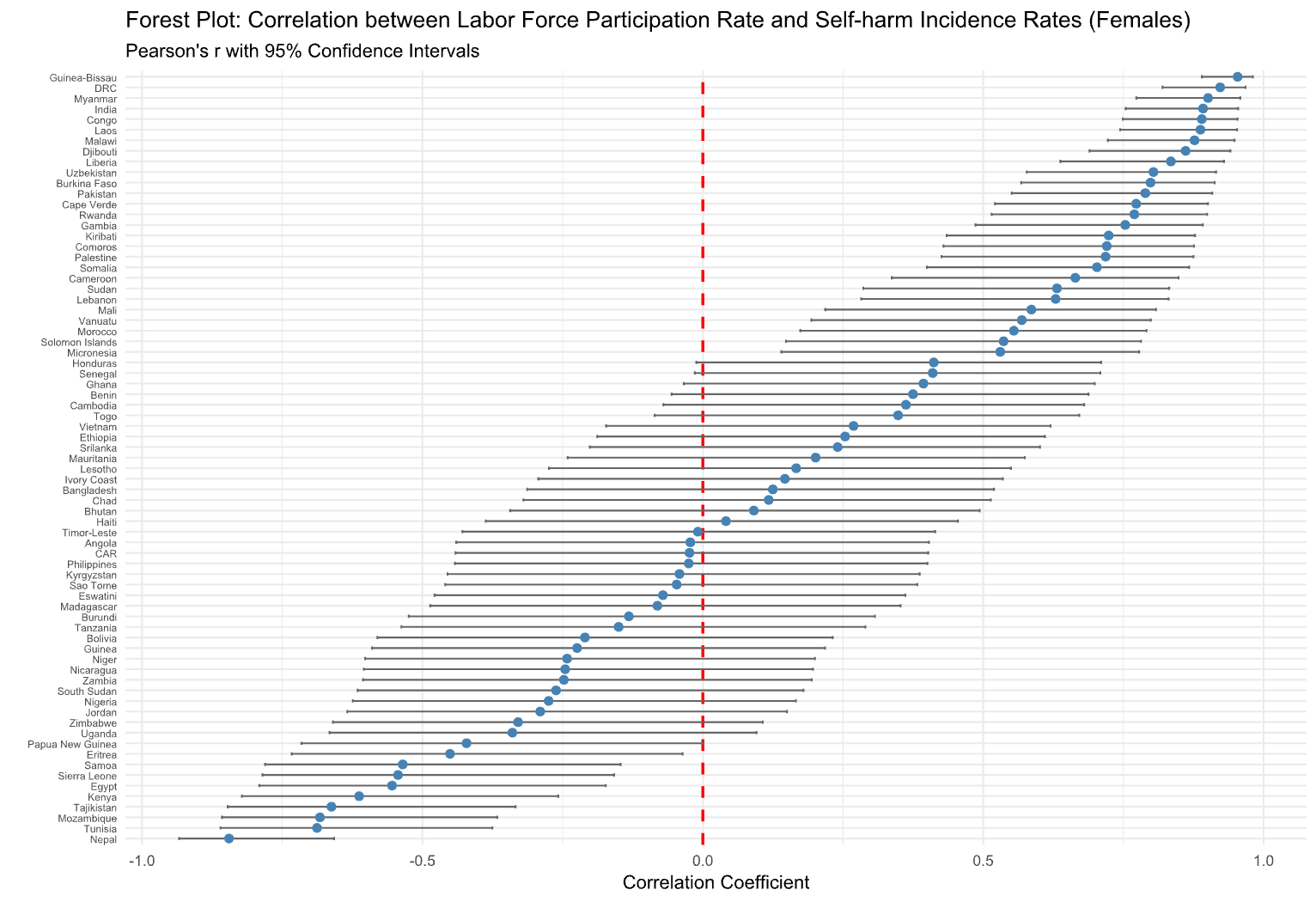
Labor Force Participation
6.
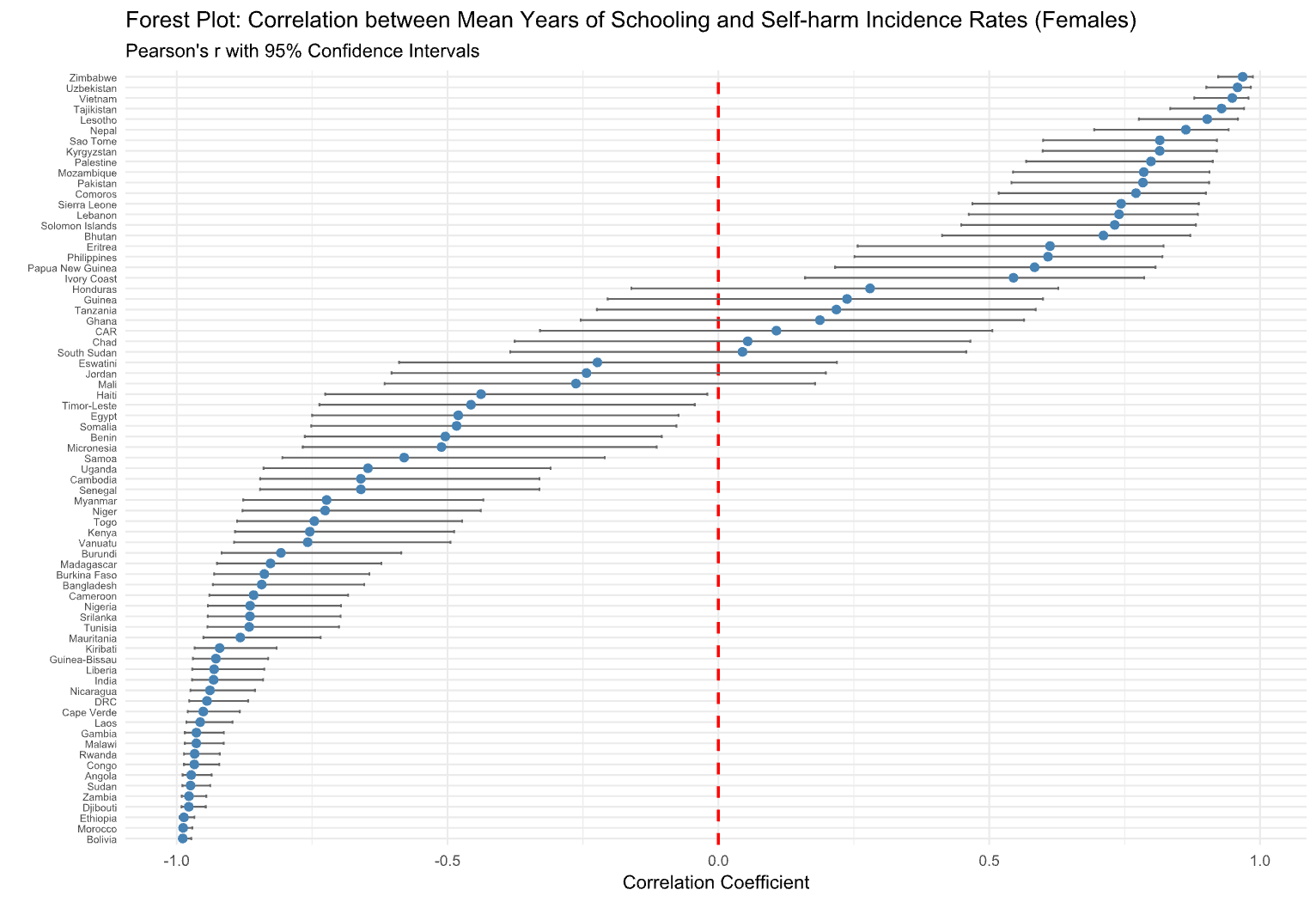
Mean Years of Schooling
7. Regulatory Quality


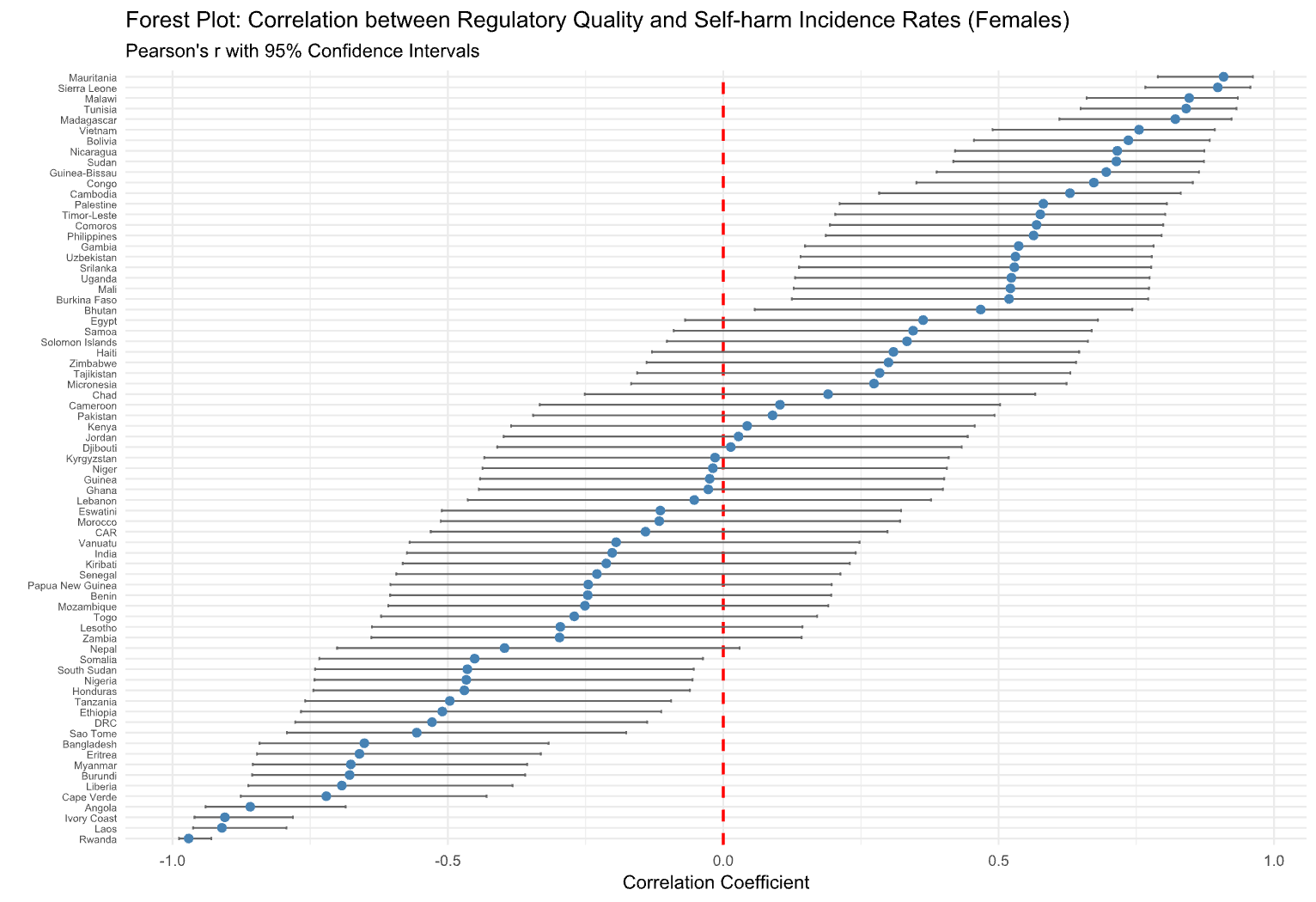


1.
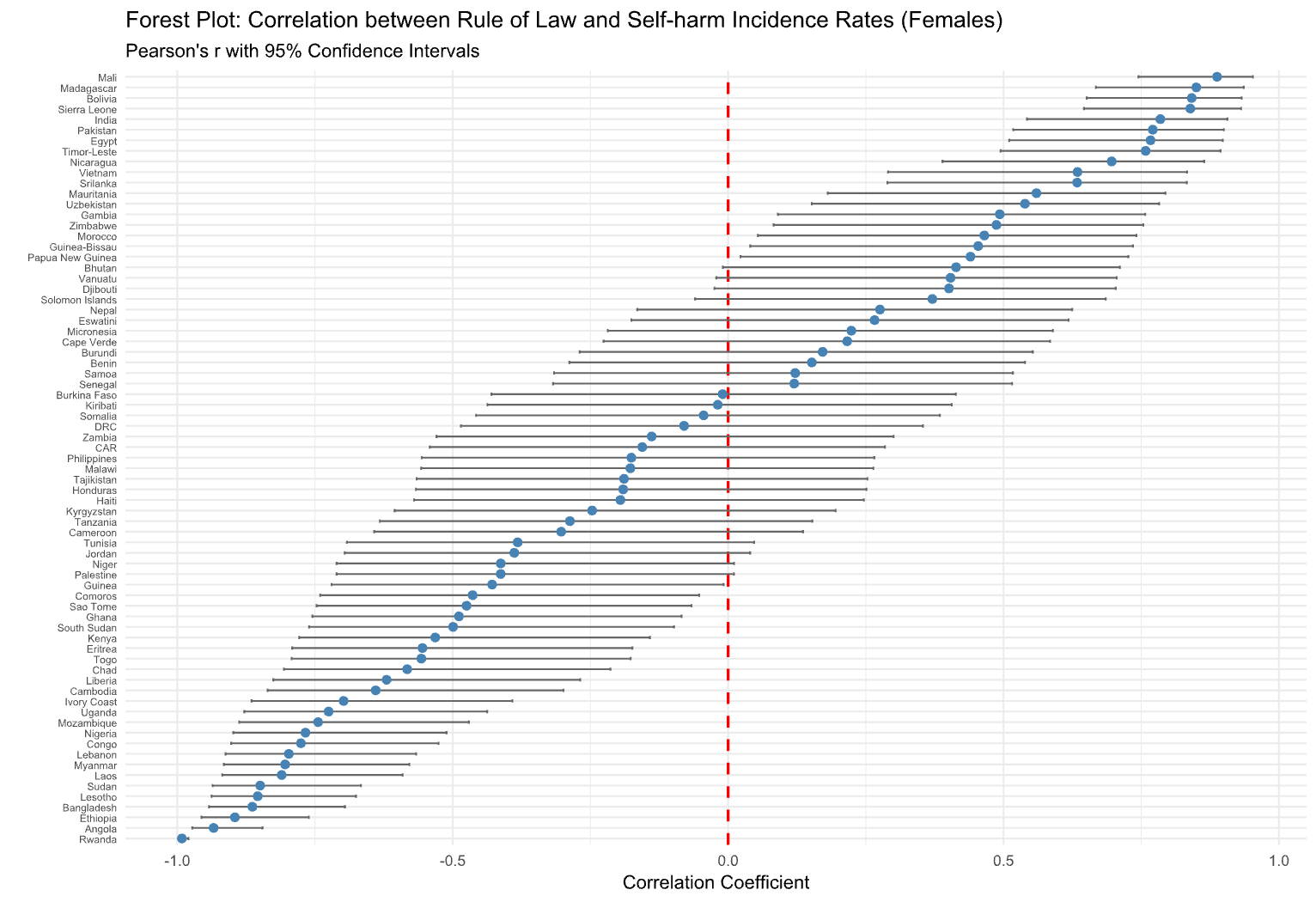
Rule of Law
2. Sociodemographic Index


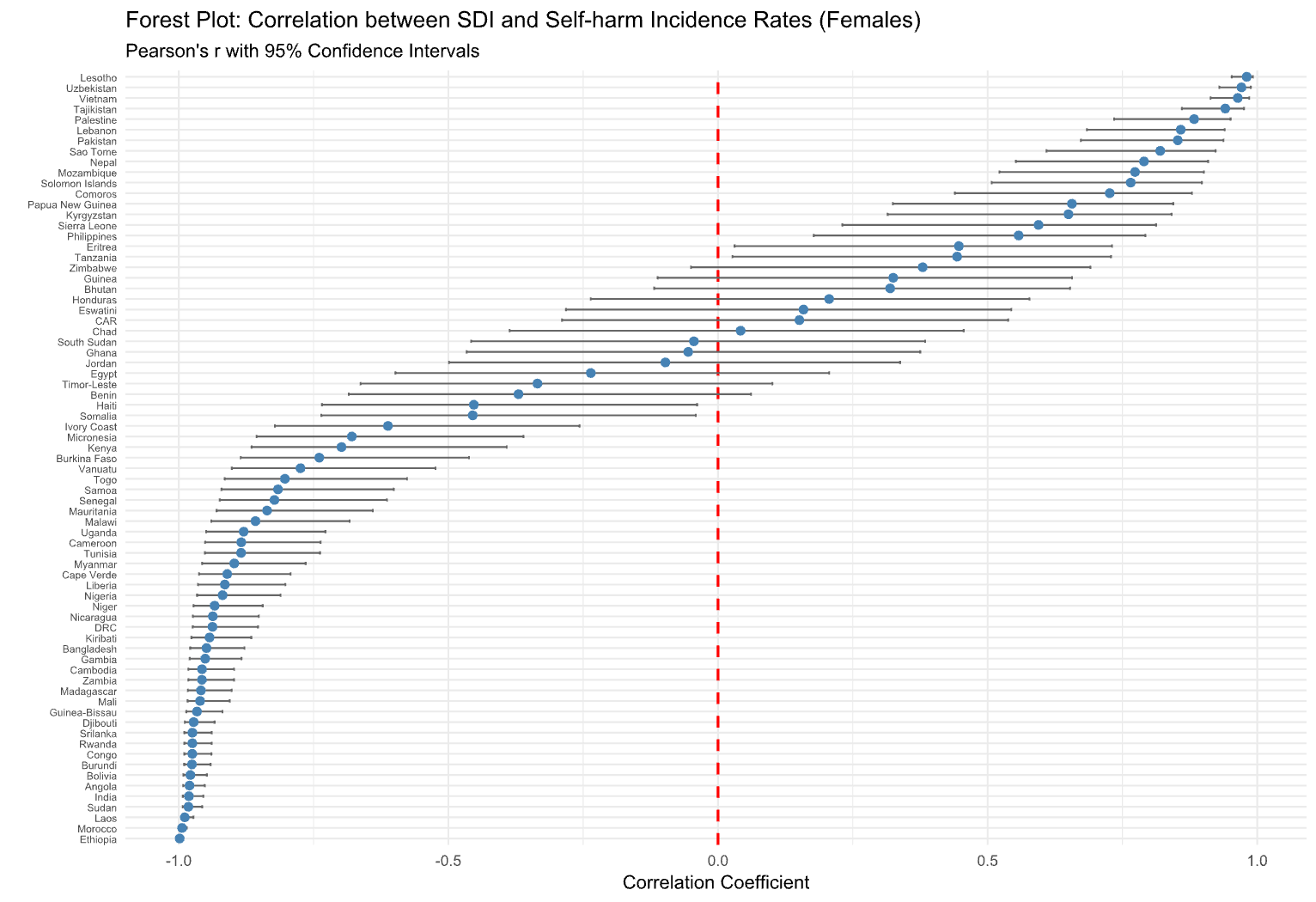


1. SEV Tobacco Use


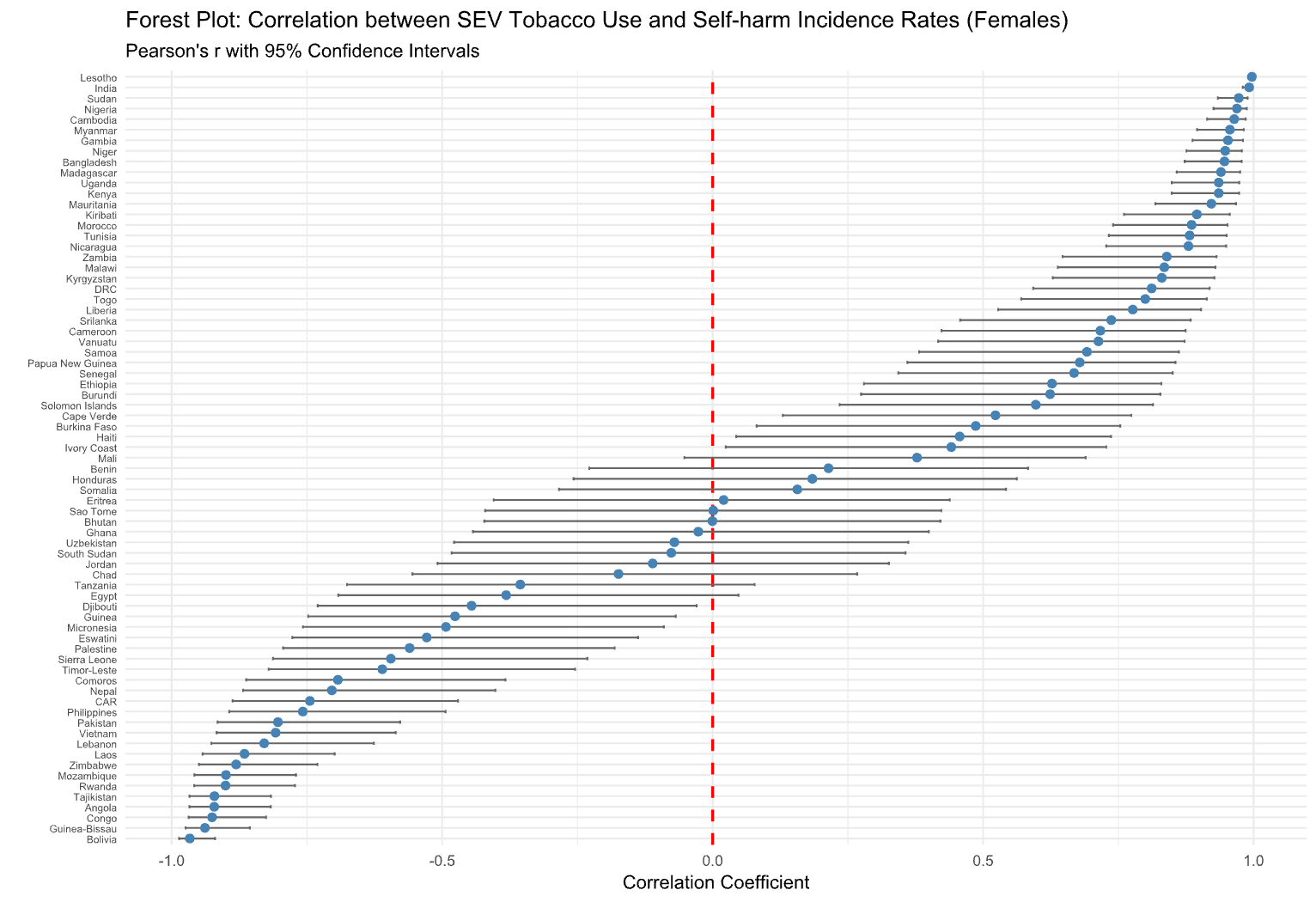


## Figure S2: Forest Plots indicating correlations between Variables in the Final model and Self-Harm Incidence Rates Among Male Adolescents.

1. Adolescent Fertility Rate


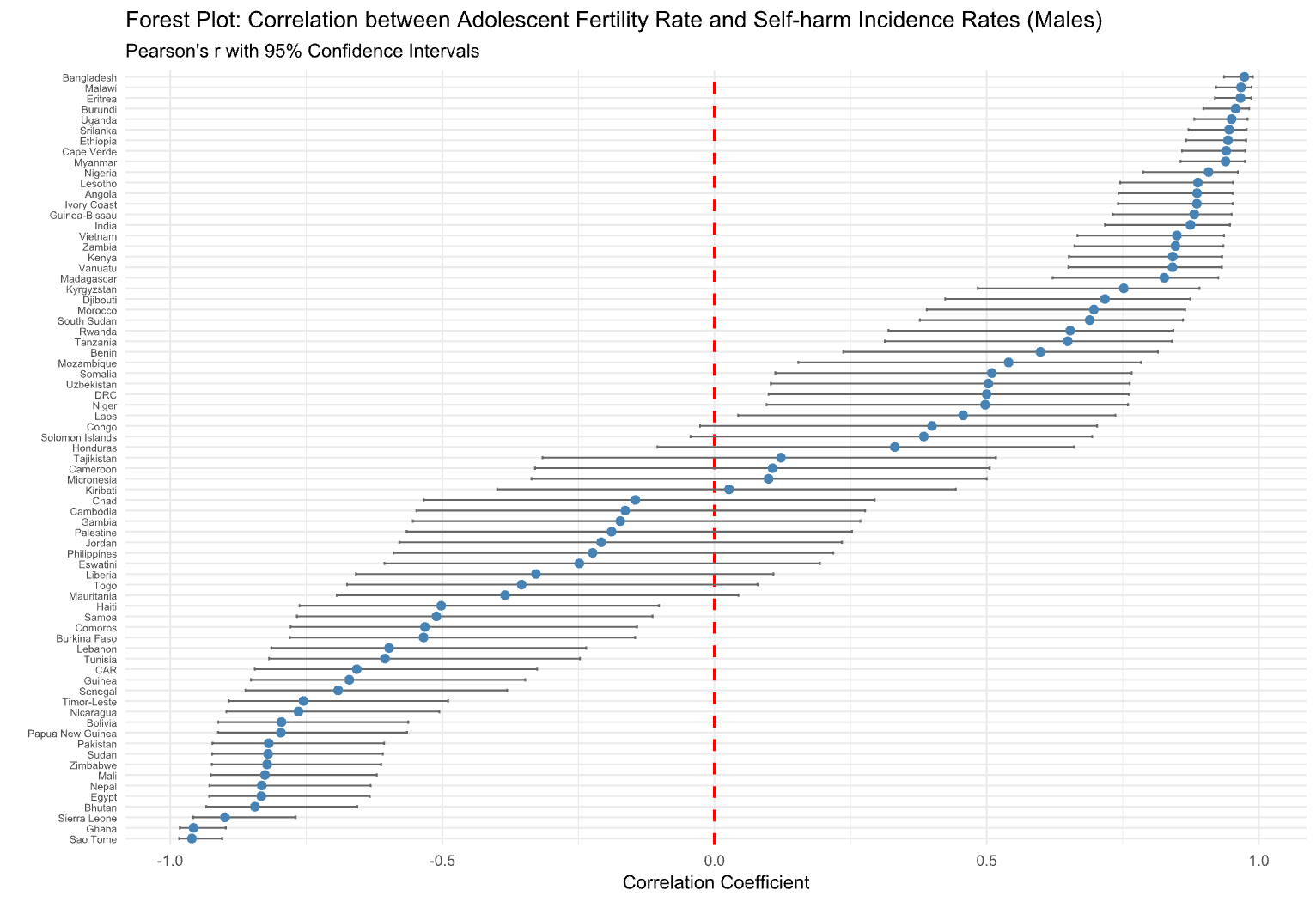


1.
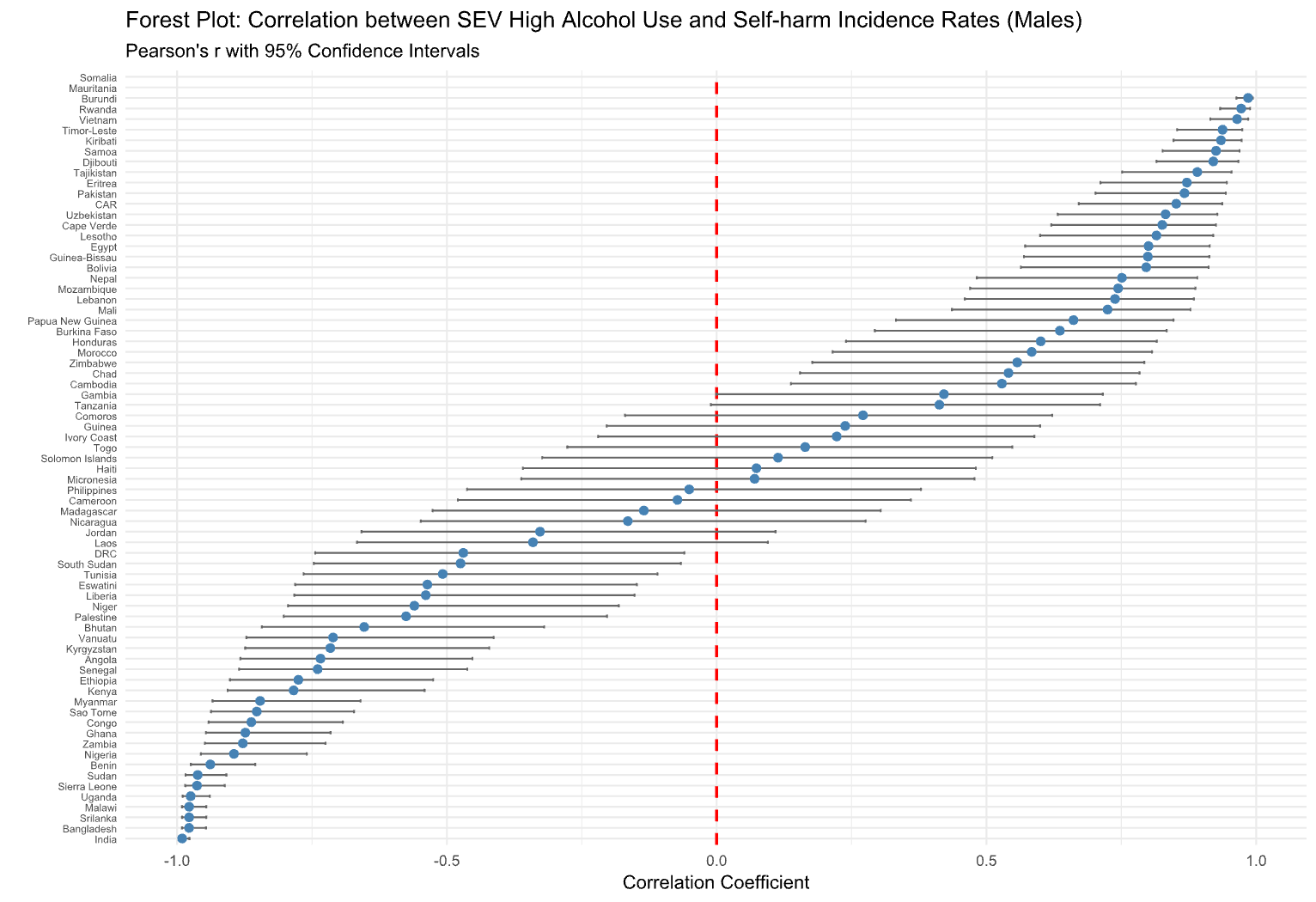
SEV High Alcohol Use
2. Control of Corruption
3.
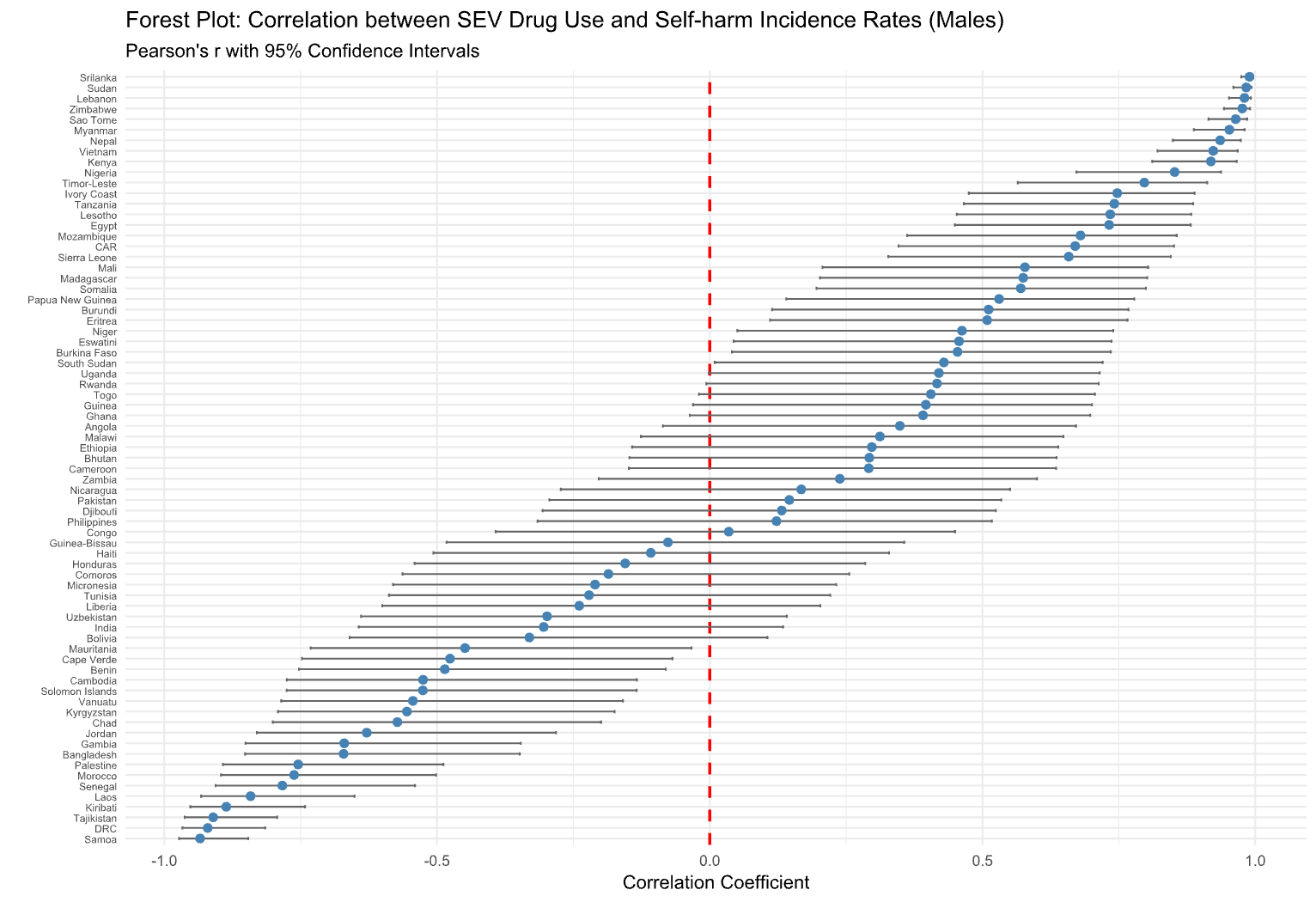

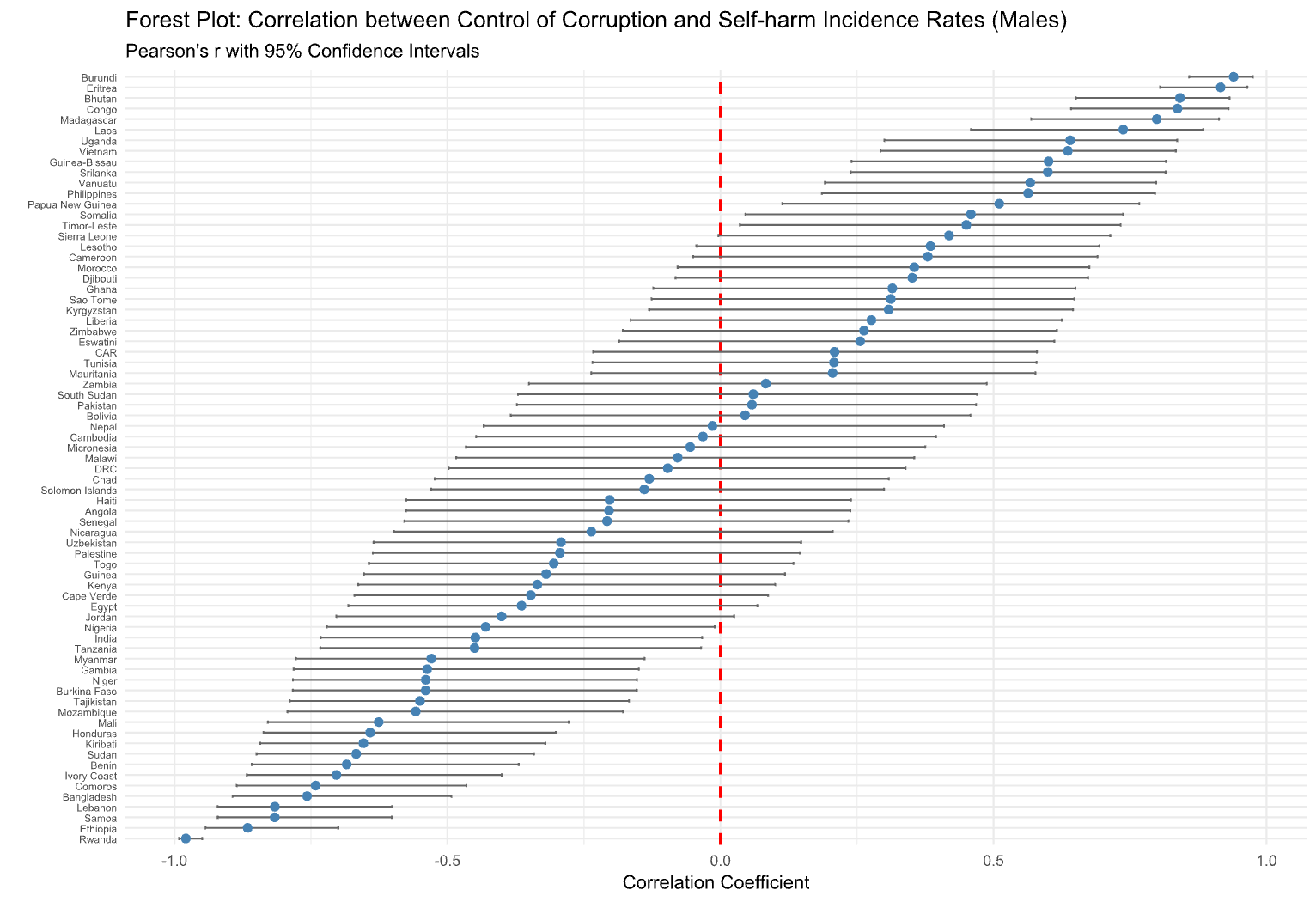
SEV Drug use
4. Young people Newly Affected with HIV


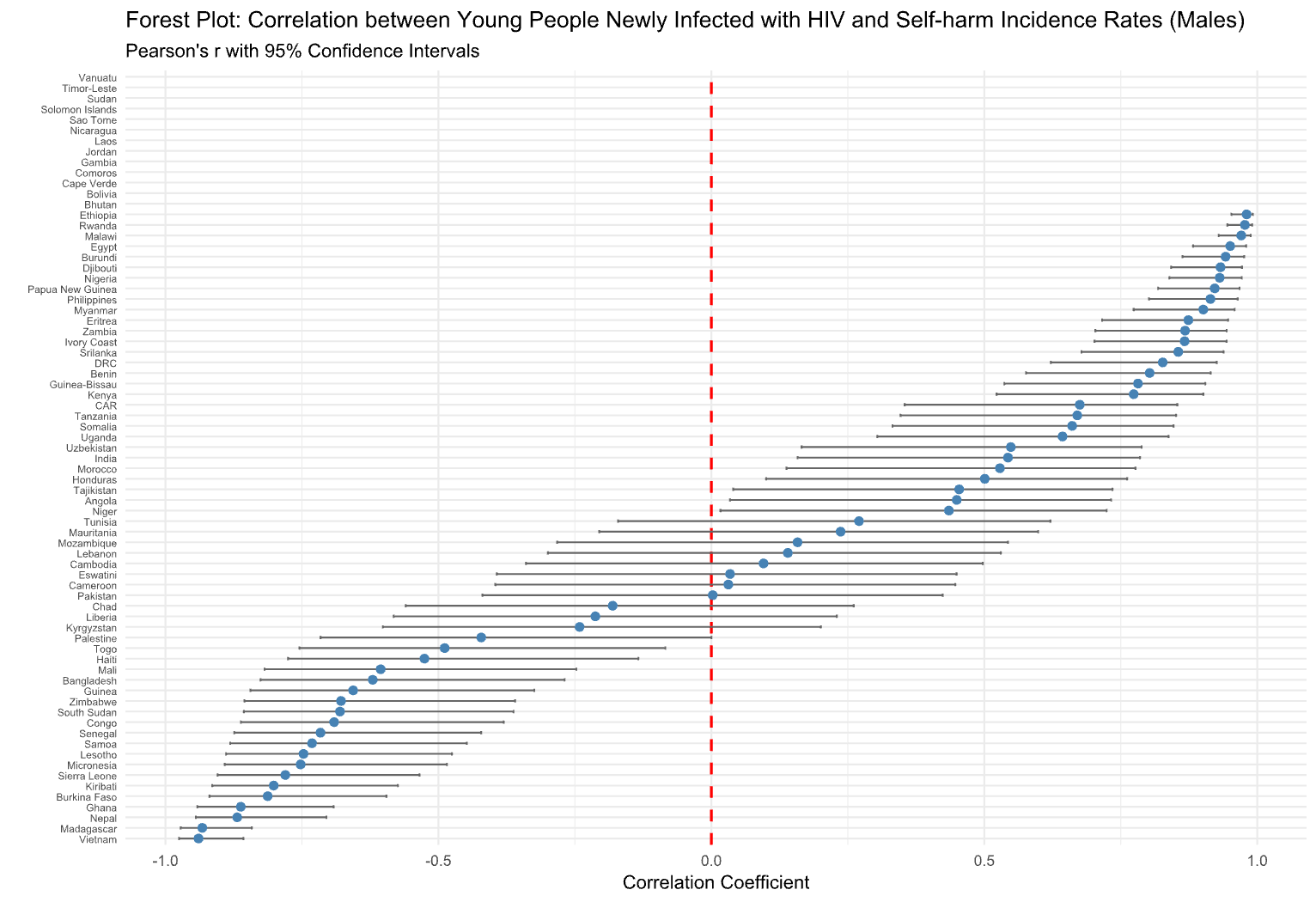


1.
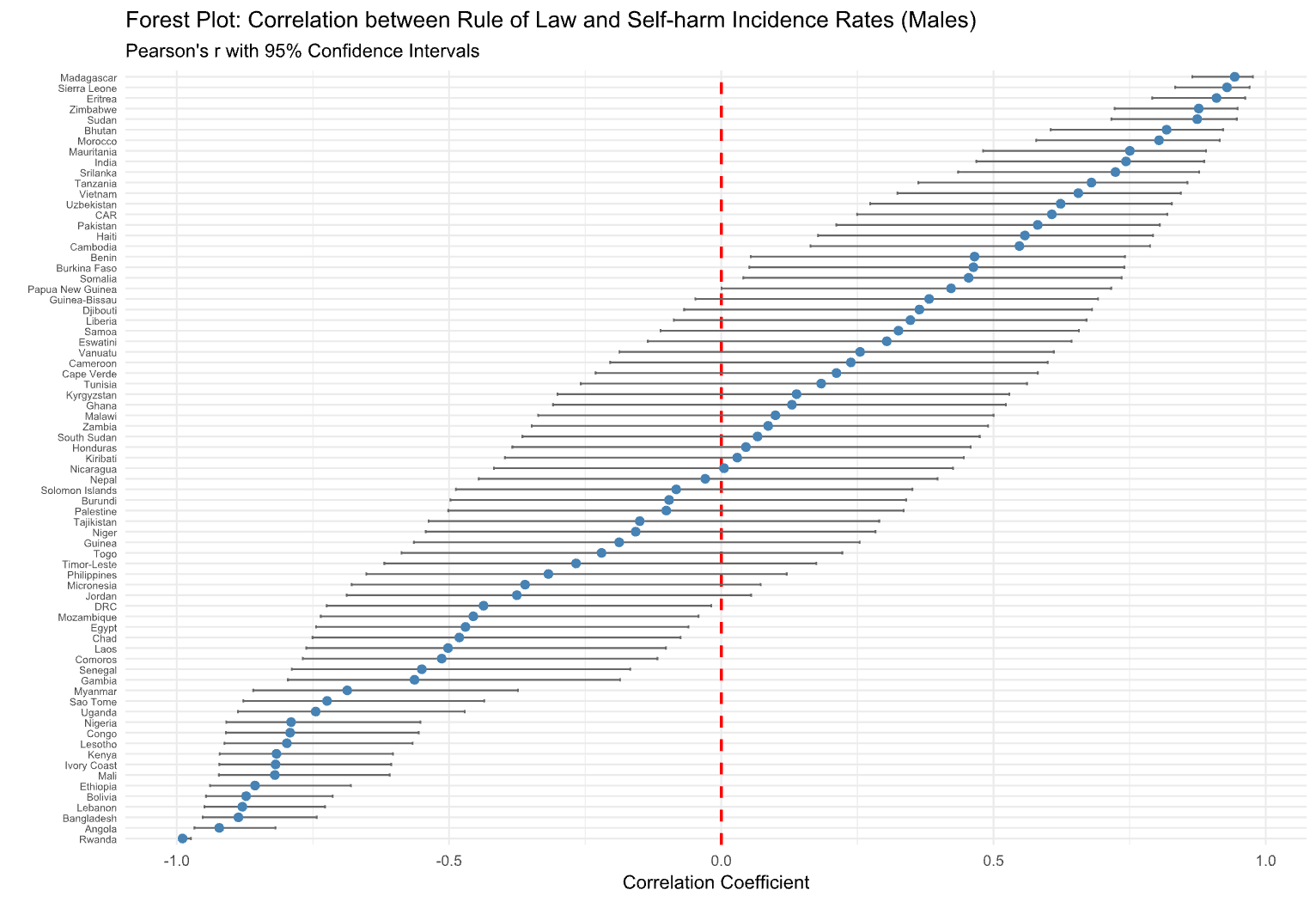
Rule of Law
2.
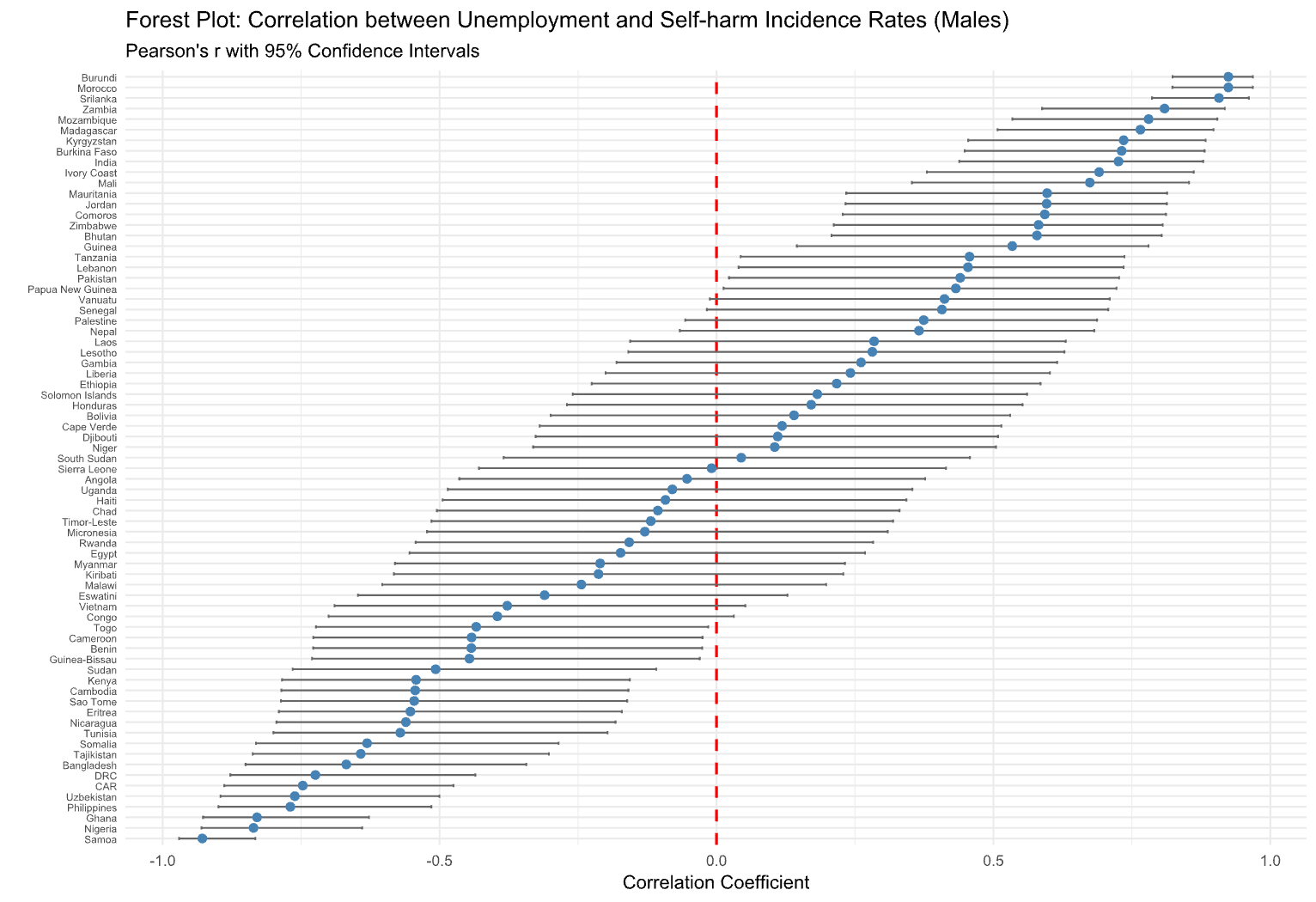
Unemployment
3. Urban Population


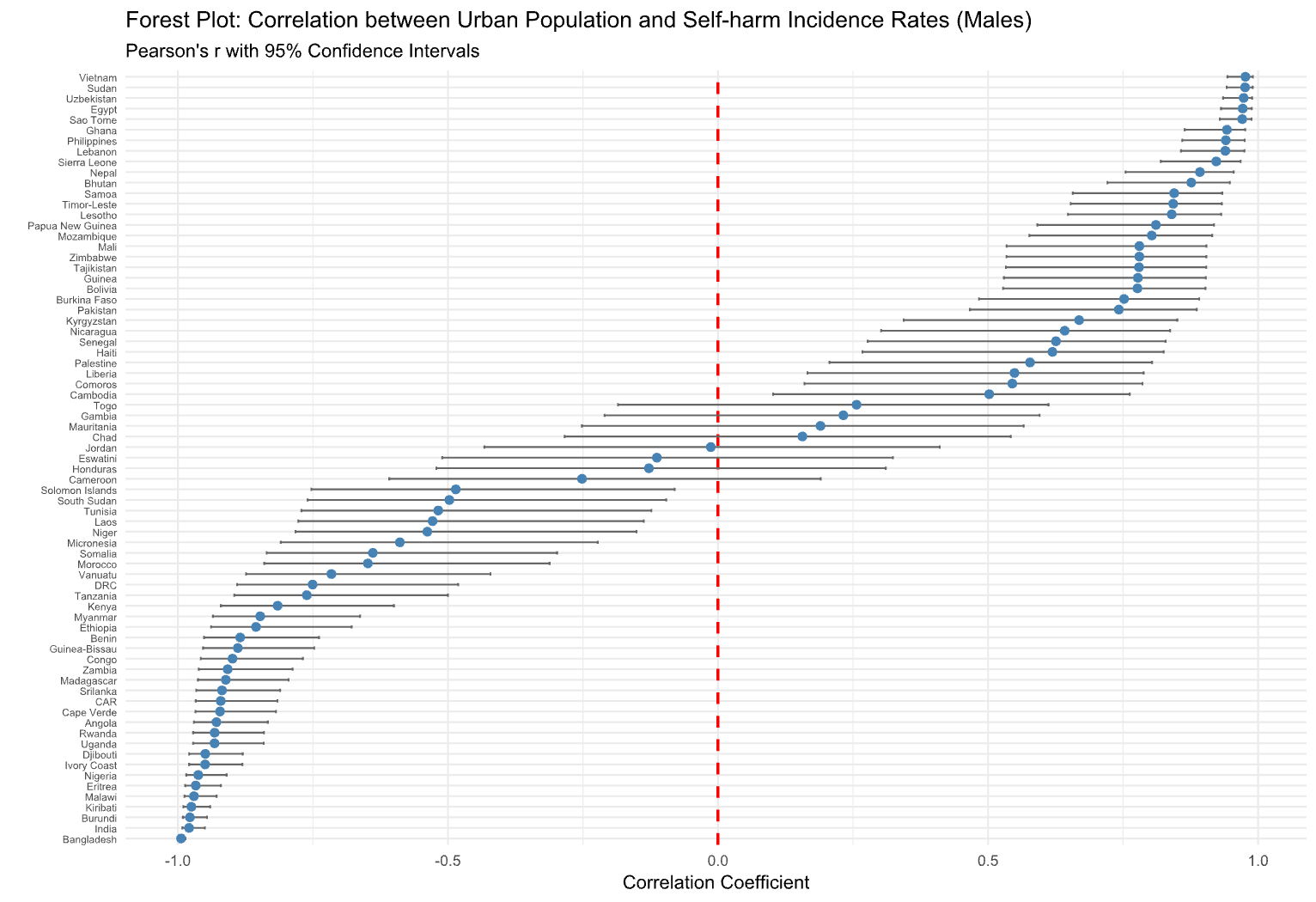


## Figure S3: Regional analysis using two-way fixed effects

1.
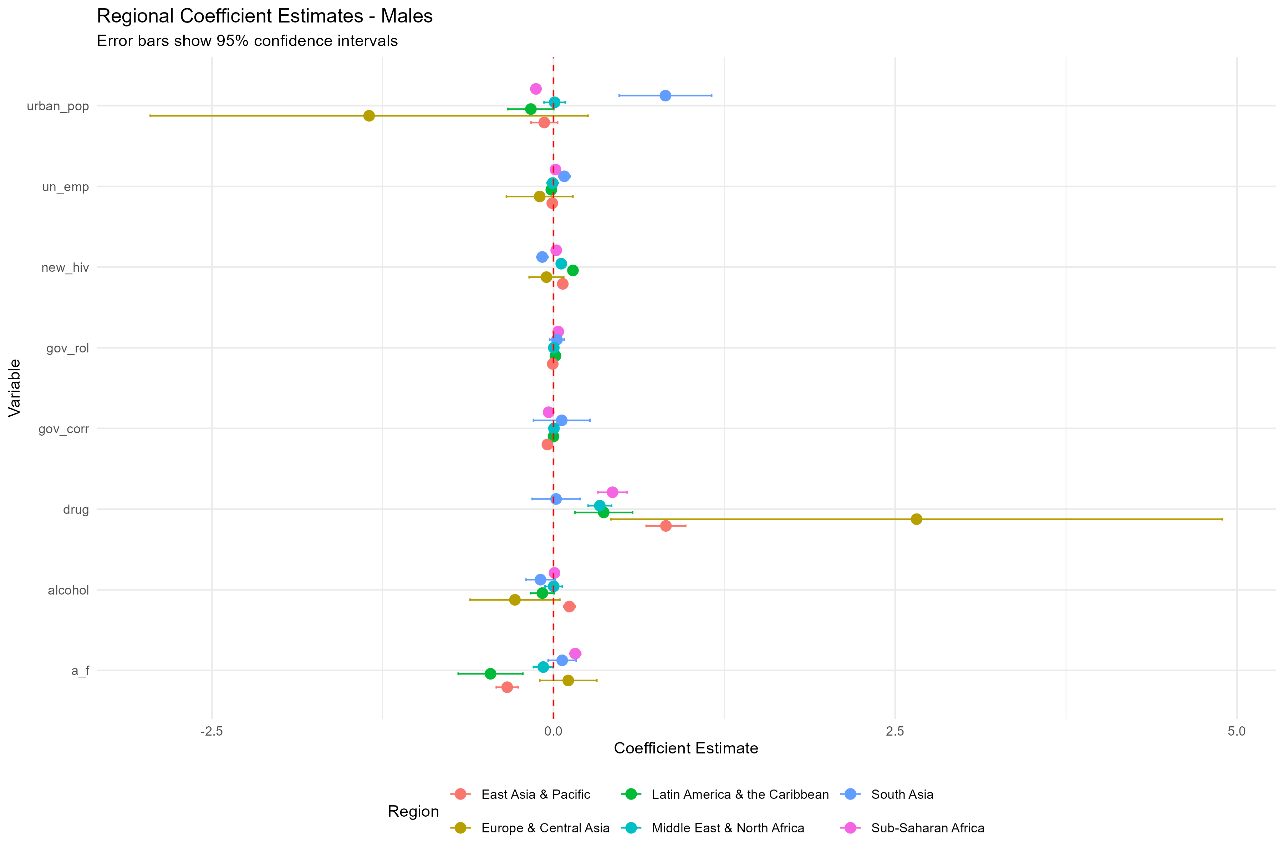
Males
2. Females
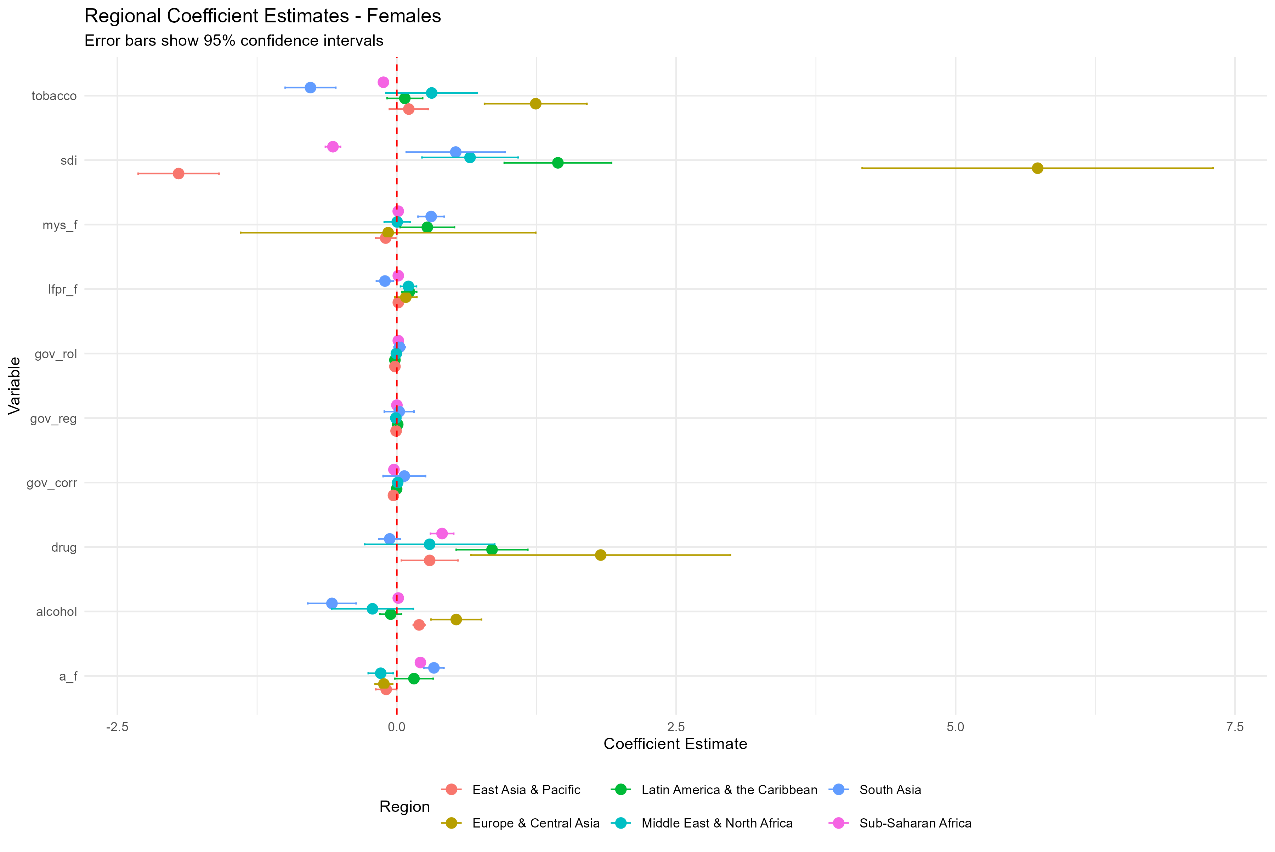


# References

**Batty GD, Whitley E, Kivimäki M, Tynelius P and Rasmussen F** (2010) Body Mass Index and Attempted Suicide: Cohort Study of 1,133,019 Swedish Men. *American Journal of Epidemiology* **172**(8)**,** 890-899. <https://doi.org/10.1093/aje/kwq274>.

**Claveria O** (2022) Global economic uncertainty and suicide: Worldwide evidence. *Social Science & Medicine* **305,** 115041. <https://doi.org/10.1016/j.socscimed.2022.115041>

**Davis Weaver N, Bertolacci GJ, Rosenblad E, Ghoba S, Cunningham M, Ikuta KS, Moberg ME, Mougin V, Han C, Wool EE, Abate YH, Adewuyi HO, Adnani QES, Adzigbli LA, Afolabi AA, Agampodi SB, Ahinkorah BO, Ahmad A, Ahmad D, Ahmad S, Ahmed A, Ahmed H, Al Hamad H, Al-Ajlouni Y, Al-amer RM, Albashtawy M, Aldhaleei WA, Ali SS, Ali W, Alomari MA, Alsabri MA, Alvis-Guzman N, Al-Worafi YM, Amindarolzarbi A, Amiri S, Andrei T, Anvari S, Arabloo J, Areda D, Artamonov AA, Ashraf T, Athari SS, Atout MMdW, Azzam AY, Badiye AD, Baghcheghi N, Bahramian S, Banach M, Barker-Collo SL, Bärnighausen TW, Barrow A, Bashiri A, Bashiru HA, Bastan M-M, Batra K, Batra R, Bayati M, Benjet C, Benzian H, Bertuccio P, Bhagavathula AS, Bhattacharjee P, Bills CB, Boppana SH, Borges G, Borhany H, Bustanji Y, Caetano dos Santos FL, Castelpietra G, Caye A, Cenderadewi M, Chandika RM, Chandrasekar EK, Charalampous P, Chen Y, Chimoriya R, Chopra H, Choudhari SG, Chu D-T, Chukwu IS, Chutiyami M, Cowden RG, Dachew BA, Dadras O, Dai X, Dalal K, Dandona L, Dandona R, Darcho SD, Darvishi Cheshmeh Soltani R, Dávila-Cervantes CA, de la Torre-Luque A, Debopadhaya S, Degenhardt L, Delgado-Enciso I, Dervišević E, Diaz MJ, Dongarwar D, Doshi OP, Dsouza HL, Dumith SC, Duraisamy S, Eboreime E, Efendi F, Ekholuenetale M, El Arab RA, Elhadi M, Elnahas G, Eltaha C, Emdadul Haque SE, Eskandarieh S, Fahim A, Faro A, Fatehizadeh A, Fazeli P, Feizkhah A, Fekadu G, Ferreira N, Fischer F, Franklin RC, Fridayani NKY, Gajdács M, Gandhi AP, Ganesan B, Gebregergis MW, Gebrehiwot M, Gebremeskel TG, Getie M, Ghadimi DJ, Ghailan KY, Ghashghaee A, Gholamrezanezhad A, Goleij P, Grada A, Grivna M, Guan S-Y, Gulati S, Gupta S, Gutiérrez RA, Gutiérrez-Murillo RS, Hamilton EB, Hanifi N, Hasan I, Hassan Zadeh Tabatabaei MS, Hay SI, Heidari M, Hemmati M, Hoan NQ, Hosseinzadeh M, Hostiuc S, Huang J, Huynh H-H, Ibitoye SE, Ilesanmi OS, Ilic IM, Ilic MD, Immurana M, Inok A, Iwu CD, Jahrami H, Jaka S, Jalilzadeh Yengejeh R, Ji Z, Jin S, Joseph N, Joshua CE, Jozwiak JJ, Kabir Z, Kadashetti V, Kanmodi KK, Kantar RS, Kapoor N, Karaye IM, Karmakar S, Kaur H, Kerr JA, Khajuria H, Khan A, Khatab K, Kheirallah KA, Kim K, Kim MS, Km Shivakumar SKM, Kolahi A-A, Koohestani HR, Krishna V, Kugbey N, Kulimbet M, Kumar GA, Kumar M, Kundu S, Kytö V, Landires I, Le NHH, Lee DW, Lee W-C, Lee YH, Lim SS, Lin J, Liu RT, López-Gil JF, Lucchetti G, Ma ZF, Maled V, Malhotra K, Malik AA, Marconi AM, Martinez-Piedra R, Marzo RR, Mathangasinghe Y, Maulik PK, Meles HN, Menezes RG, Meretoja TJ, Mestrovic T, Michalek IM, Miller TR, Mirza M, Misganaw A, Mittal C, Mohamed AZ, Mohamed NS, Mohammadian-Hafshejani A, Mokdad AH, Molinaro S, Monasta L, Moodi Ghalibaf A, Morrison SD, Motappa R, Mughal F, Mulita F, Munkhsaikhan Y, Murray CJL, Muthu S, Myung W, Nafei A, Naghavi P, Naik GR, Naik G, Natto ZS, Naveed M, Navid S, Nayak BP, Nazri-Panjaki A, Netsere HB, Neupane SP, Nguyen HAH, Nguyen NNY, Nguyen PT, Nguyen PT, Nguyen VT, Nikoobar A, Noguer I, Nomura S, Nri-Ezedi CA, Nuñez-Samudio V, Nzoputam OJ, Oancea B, Oduro MS, Oh I-H, Okeke SR, Oluwafemi YD, Ong SK, Ordak M, Orpana HM, Ortiz-Prado E, Osuagwu UL, Padron-Monedero A, Padubidri JR, Palma-Alvarez RF, Pandey A, Pandey A, Pantazopoulos I, Park S, Park S, Pashaei A, Patel J, Pawar S, Peprah P, Peres MFP, Petcu I-R, Philip AK, Phillips MR, Piracha ZZ, Pradhan J, Prates EJS, Pribadi DRA, Puvvula J, Qattea I, Qian G, Radhakrishnan V, Raghav P, Rahimibarghani S, Rahimi-Movaghar A, Rahimi-Movaghar V, Rahman MM, Rahman M, Rahman MA, Rahmanian M, Rajpoot PL, Ramadan MM, Ramasamy SK, Rani S, Rao M, Rao SJ, Rashidi M-M, Rastogi P, Rathish D, Rawaf DL, Reifels L, Rezaeian M, Rhee TG, Rickard J, Roever L, Rony MKK, S N C, Saddik BA, Sadeghian F, Saeb MR, Saeed U, Saeedi Moghaddam S, Safari M, Sagoe D, Saheb Sharif-Askari N, Sahoo PM, Sahoo SS, Salamati P, Salihu D, Salimi S, Salum GA, Sameen S, Samy AM, Santric-Milicevic MM, Sarkar C, Sarode GS, Sarode SC, Sathian B, Schumacher AE, Šekerija M, Semreen MH, Sepanlou SG, Shafie M, Shahid S, Shaikh A, Shaikh MA, Sharifan A, Sharifi Rad J, Sharma A, Sharma V, Sheikhi RA, Shetty M, Shetty PH, Shetty PK, Shivarov V, Shool S, Singh P, Singh P, Singh S, Socea B, Stein DJ, Stein MB, Sun J, Swain CK, Szarpak L, T Y SS, Tabatabaei SM, Tabche C, Tareke M, Temsah M-H, Thum CC, Tiruye TY, Tovani-Palone MR, Tran NM, Tran TH, Tran Minh Duc N, Tromans SJ, Truyen TTTT, Tsegay GM, Tumurkhuu M, Vahdati S, Vaithinathan AG, Valdez PR, Vasankari TJ, Veroux M, Verras G-I, Vinayak M, Vos T, Walde MT, Wang Y, Ward JLL, Wickramasinghe ND, Wojewodzic MW, Yesodharan R, Yiğit A, Yin D, Yip P, Yon DK, Yonemoto N, Yu C, Zare I, Zeariya MGM, Zhang H, Zhong CC, Zhu B, Zhumagaliuly A and Naghavi M** (2025) Global, regional, and national burden of suicide, 1990&#x2013;2021: a systematic analysis for the Global Burden of Disease Study 2021. *The Lancet Public Health* **10**(3)**,** e189-e202. <https://doi.org/10.1016/S2468-2667(25)00006-4>.

**Er ST, Demir E and Sari E** (2023) Suicide and economic uncertainty: New findings in a global setting. *Ssm-Population Health* **22,** 101387. <https://doi.org/10.1016/j.ssmph.2023.101387>

**Klonsky ED and Moyer A** (2008) Childhood sexual abuse and non-suicidal self-injury: meta-analysis. *The British Journal of Psychiatry* **192**(3)**,** 166-170.

**Lange S, Cayetano C, Jiang H, Tausch A and e Souza RO** (2023) Contextual factors associated with country-level suicide mortality in the Americas, 2000–2019: a cross-sectional ecological study. *The Lancet Regional Health–Americas* **20**. <https://doi.org/10.1016/j.lana.2023.100450>.

**Lari MS and Sefiddashti SE** (2023) Socio-economic, health and environmental factors influencing suicide rates: A cross-country study in the Eastern Mediterranean region. *Journal of Forensic and Legal Medicine* **93,** 102463. <https://doi.org/10.1016/j.jflm.2022.102463>

**Lyu J, Ding L, Zhou Z, Zhang Q and Li X** (2025) The Correlation between Economic Development and Suicide Rate-Based on Global WHO and WB database. *Frontiers in Public Health* **13,** 1596682. <https://doi.org/10.3389/fpubh.2025.1596682>

**Obama FXN** (2025) Gender inequality, women’s human capital and female suicide in selected MENA countries. *Ssm-Population Health***,** 101819. <https://doi.org/10.1016/j.ssmph.2025.101819>

**Rajkumar RP** (2023) The association between nation-level social and economic indices and suicide rates: A pilot study. *Frontiers in Sociology* **8,** 1123284. <https://doi.org/10.3389/fsoc.2023.1123284>.

**Runkle JR, Harden S, Hart L, Moreno C, Michael K and Sugg MM** (2023) Socioenvironmental drivers of adolescent suicide in the United States: A scoping review. *Journal of rural mental health* **47**(2)**,** 65. <https://doi.org/10.1037/rmh0000208>

**Yan N, Luo Y, Mackay LE, Wang Y, Wang Y, Wang Y, Shiferaw BD, Wang J, Tang J and Yan W** (2024) Global patterns and trends of suicide mortality and years of life lost among adolescents and young adults from 1990 to 2021: a systematic analysis for the Global Burden of Disease Study 2021. *Epidemiology and Psychiatric Sciences* **33,** e52. <https://doi.org/10.1017/s2045796024000532>.

**Ye Z, Chen M, Wang X, Xie Z, Zhang D, Wu D, Zhao Y, Qu Y and Jiang Y** (2025) Examining the association between tobacco and its substitutes use with psychosocial symptoms among 187,329 adolescents: A comparative analysis across 47 countries with varied universal health coverage index. *Global Transitions* **7,** 128-135. <https://doi.org/https://doi.org/10.1016/j.glt.2025.02.007>.

**Zhou X, Li R, Cheng P, Wang X, Gao Q and Zhu H** (2024) Global burden of self-harm and interpersonal violence and influencing factors study 1990–2019: analysis of the global burden of disease study. *BMC public health* **24**(1)**,** 1035.
