# Supplementary material for: The trend of self-harm incidence rates among adolescents in low- and lower-middle-income countries and its associated contextual factors
Source: Glob Ment Health (Camb). 2026 Jun 2;13:e142. doi: 10.1017/gmh.2026.10241 (PMC13373272; doi:10.1017/gmh.2026.10241)
Supplement: Peer review history

# Reviewer report: Review: The trend of self-harm incidence rates among adolescents in low- and lower-middle-income countries and its associated contextual factors — R0/PR2

**DOI:** 10.1017/gmh.2026.10241.pr2

Page 9 of 27, line 11, reductio to reduction.

# Reviewer report: Review: The trend of self-harm incidence rates among adolescents in low- and lower-middle-income countries and its associated contextual factors — R0/PR3

**DOI:** 10.1017/gmh.2026.10241.pr3

1. SUMMARY OPINION

1.1 This manuscript examines trends in adolescent self-harm incidence across 77 low- and lower-middle-income countries (LLMICs) from 2000-2021 and investigates contextual factors associated with these trends using GBD 2021 data. The topic addresses a significant gap in the literature on suicide and self-harm in resource-limited settings, and the sex-specific analysis is a strength. However, the manuscript requires substantial revisions before publication consideration.

1.2 Major Strengths:

-Addresses an important and understudied area (self-harm in LLMICs);

-Comprehensive coverage (77 countries, 22 years);

-Sex-specific analysis revealing differential patterns;

-Appropriate statistical methods (joinpoint regression, fixed-effects modeling);

-Use of internationally comparable data sources.

1.3 Major Limitations:

-To my knowledge, GBD 2023 data has been released and is downloadable. Why doesn’t the author consider using the latest GBD 2023 data?

-Over-reliance on modeled GBD estimates with questionable reliability in low-data settings;

-Missing data management potentially introduces bias despite imputation efforts;

-Limited recent literature (lacks sufficient 2024-2025 references despite submission date);

- fallacy concerns inadequately addressed;

-Weak theoretical framework linking contextual factors to self-harm;

-Insufficient discussion of causality vs. association;

-Language imprecision and non-standard terminology in places;

-Limited exploration of mechanisms underlying observed associations;

1.4 Statistical Issues:

-Low R-squared values (0.165 males, 0.232 females) suggest substantial unexplained variance;

-Potential multicollinearity despite VIF screening;

-Country and year fixed effects may over-control for relevant variation;

1.5 Language and Presentation:

-Generally acceptable English but requires copyediting;

-Some awkward phrasing and grammatical inconsistencies;

-Abbreviations occasionally used inconsistently;

1.6 Literature Review:

-Lacks recent 2024-2025 high-impact publications;

-Insufficient engagement with theoretical frameworks in global mental health;

-Missing key regional studies from LLMICs;

1.7 Decision Recommendation: Major Revision

DETAILED COMMENTS

2. INTRODUCTION

2.1 Conceptual Clarity and Definitions

-The manuscript conflates “self-harm” and “suicide” without adequately distinguishing between them. While the authors acknowledge self-harm as a risk factor for suicide (lines 5-7), the introduction oscillates between discussing suicide mortality and self-harm without clear conceptual boundaries.

-“Recently, adolescent suicide and self-harm have gained significant attention” treats them as parallel phenomena without acknowledging that self-harm includes both suicidal and non-suicidal acts.

-States “self-harm often begins in adolescence” citing Bolger et al. 1989, but this citation discusses “onset of suicidal ideation,” not self-harm behavior.

-The GBD definition of self-harm (Methods section) includes ICD codes for intentional self-harm resulting in death or injury, which may exclude non-suicidal self-injury (NSSI)—this limitation should be acknowledged in the Introduction.

2.2 Literature Review and Contextualization

-Insufficient Recent Literature: Lack of sufficient 2024-2025 citations, with only one 2025 reference (Tan et al.).

-Theoretical Framework Inadequately Developed: Lines 15-22 list various contextual factors without providing a coherent theoretical framework. The reference to Lund et al. 2018’s conceptual framework (line 23) is mentioned but not elaborated.

-Gap Statement Lacks Precision: Lines 24-28 state that studies on contextual factors are “scarce” and “most focus on high income countries,” but provide only two citations (Iemmi et al. 2016; Lange et al. 2023). This is insufficient to establish a clear research gap. Recommendation: Conduct a more systematic review of existing literature. Provide specific numbers: “Of X studies examining contextual factors and suicide/self-harm, only Y focused on LMICs, and none examined sex-specific patterns in adolescents.” Cite relevant systematic reviews. Explain why LLMICs specifically warrant separate analysis (different economic structures, governance challenges, data limitations, etc.).

2.3 Logic and Coherence

Recommendation: Restructure for logical flow:

-Global burden and significance of self-harm/suicide;

-Adolescent-specific patterns and why this population matters;

-Known individual-level risk factors (briefly);

-Emerging evidence on population-level contextual factors (with theoretical framework);

-Critical gap: lack of LLMIC-focused, sex-specific research on contextual factors

-Study aims with clear hypotheses.

2.4 Specific Comments

-Line 2: “720,000 individuals die by suicide each year”—clarify this is a global estimate for all ages; adolescent-specific figures should follow.

-Line 3: “73% of these deaths occurring in low- and middle-income countries”—specify whether this is LMICs combined or specifically LLMICs (which are a subset).

-Line 5-6: “increases the risk of suicide by more than 30 times”—this figure requires context. Is this compared to general population? Over what time period? The cited Hawton et al. 2015 study was UK-based; extrapolation to LLMICs is questionable.

-Line 10: “suicide is the third leading cause of death among young people”—specify age range and whether this is globally or specific regions.

-Lines 11-14: The discussion of declining trends contradicts the later statement (lines 13-14) about regions where declines were “not observed.” This creates confusion about whether trends are positive or concerning. Clarify: “While global trends show decline, significant regional heterogeneity exists, with certain LLMIC regions experiencing increases.”

-Line 17: “suicidal behavior and self-harm has been linked”—grammatical error; should be “have been linked.”

-Line 19-20: The list of factors is too long and unstructured. Group conceptually: “socioeconomic factors (poverty, inequality, health expenditure), health system factors (practitioner ratios), and substance-related factors (alcohol, drugs).”

-Lines 24-25: “However, studies examining suicide and self-harm, especially among adolescents, are scarce”—this contradicts the earlier extensive literature review. Clarify: studies exist but few examine POPULATION-LEVEL contextual factors in LLMICs with sex-specific analyses.

-Lines 28-30: Study aims are clearly stated but lack specificity. Add: “We hypothesized that socioeconomic development, governance quality, and reproductive health indicators would show sex-specific associations with self-harm incidence.”

3. METHODS

3.1 Study Design

-Line 33: “ecological study design”—Acknowledge the primary limitation upfront: ecological studies cannot infer individual-level causation (ecological fallacy). State: “This design is appropriate for generating hypotheses about population-level factors but cannot establish individual-level causality.”

3.2 Data Sources

-Problem 1: Data Quality Not Addressed

The manuscript relies entirely on GBD modeled estimates but fails to acknowledge the substantial uncertainty and data sparsity in LLMICs. Many of these countries lack robust vital registration systems, and GBD estimates rely heavily on statistical modeling and imputation.

Recommendation:

Add a paragraph acknowledging: “GBD estimates for LLMICs have varying levels of certainty depending on data availability. Countries with sparse data rely more heavily on predictive covariates and regional patterns. While GBD represents the most comprehensive available source, users should interpret country-specific estimates cautiously, particularly for countries with low data quality ratings.” Consider including GBD data quality metrics in supplementary materials.

-Problem 2: Self-Harm Definition Ambiguity

-Lines 37-39 provide ICD codes but don’t clarify what behaviors are captured. Does this include all emergency department visits? Only hospitalizations? Deaths? Non-fatal self-poisoning? Cutting? The heterogeneity in what gets medicalized and reported varies dramatically across LLMICs.

Recommendation:

Explicitly state: “GBD self-harm estimates include both fatal and non-fatal intentional self-injury events that resulted in healthcare contact or death. These estimates likely underestimate true incidence as many self-harm events, particularly in LLMICs, do not result in formal healthcare contact due to stigma, access barriers, and cultural factors. Additionally, the proportion of self-harm events that are medically attended varies substantially across countries, potentially introducing systematic bias into cross-country comparisons.”

-Lines 41-49 (Contextual Factors):

-Problem: Variable Selection Justification Weak

The manuscript states variables were selected based on “previous literature” (line 41) but provides only two citations (Lange et al. 2023; Rajkumar 2023). The rationale for including specific variables (e.g., “high body mass index,” “sub-optimal temperature”) is unclear.

Explain why certain variables (BMI, temperature) were considered but ultimately not included or retained.

3.3 Statistical Analysis

-Lines 51-60 (Joinpoint Regression):

-Problem: Insufficient Detail

The description is adequate for those familiar with joinpoint regression but lacks clarity for general readers.

Recommendation:

Add: “Joinpoint regression identifies points where statistically significant changes in trend occur, allowing for more nuanced understanding than simple linear trends. The AAPC represents the overall trend across the entire period, accounting for any changes in trajectory.”

-Lines 61-64 (Missing Data):

-Critical Problem: Missing Data Handling Inadequately Described and Justified

The manuscript excludes variables with >30% missingness and uses “stratified median imputation” but provides no justification for:

Why 30% threshold was chosen? Why median imputation (rather than mean, multiple imputation, or other methods)? How missingness patterns differed across countries and variables? Potential bias introduced by excluding countries (Afghanistan, North Korea, Syria, Yemen)?

-Lines 66-75 (Regression Modeling):

-Problem 1: Model Selection Process Lacks Transparency

The stepwise backward elimination approach is mentioned but:

No specification of p-value threshold for variable retention. No discussion of potential overfitting. No cross-validation or model stability assessment. No sensitivity analyses with alternative model specifications.

Recommendation:

Specify: “Variables with p<0.05 in univariate analysis were retained. Backward elimination used AIC for model comparison, removing variables sequentially until AIC no longer improved. To assess model stability, we conducted bootstrap resampling (1000 iterations) to examine coefficient stability (results in Supplementary Table X).”

-Problem 2: Fixed Effects Selection

Lines 73-75 mention Hausman test but don’t explain WHY fixed effects are preferred beyond statistical testing.

-Problem 3: Low R-squared Values Not Addressed

The Results show R²=0.165 (males) and 0.232 (females), meaning >75% of variance remains unexplained. This crucial limitation is never mentioned.

-Problem 4: No Mention of Multicollinearity Diagnostics

Line 68 mentions VIF but doesn’t specify threshold or report values.

-Problem 5: No Sensitivity Analyses Described

Essential for robust inference, especially given data quality concerns.

4. RESULTS

4.1 Trend Analysis (Lines 77-90)

-Problem 1: Lack of Contextualization

Results present AAPCs without discussing magnitude of incidence rates. A 3% annual change means very different things if baseline rate is 50/100,000 vs. 500/100,000.

Recommendation:

Begin Results with descriptive statistics: “In 2000, median self-harm incidence was X per 100,000 (IQR: X-X) among males and X per 100,000 (IQR: X-X) among females across the 77 countries. By 2021, this had changed to X and X, respectively. Substantial heterogeneity existed, with country-level rates ranging from X to X.”

-Problem 2: Inconsistent Reporting

Lines 79-83 report top 5 countries for females but lines 85-90 report different numbers for males (top 5 for decreases, top 5 for increases). Use parallel structure.

-Problem 3: Figure 1 Not Adequately Described

The figure is referenced but not interpreted. What geographic patterns exist? Do specific regions cluster?

-Problem 4: Statistical Significance of Trends Not Reported

AAPCs are presented without confidence intervals in text (though presumably in figures). Were all these trends statistically significant?

4.2 Contextual Factors Analysis

Tables 1 and 2:

-Problem 1: Effect Size Interpretation Lacking

Tables present coefficients for log-transformed variables without interpretation. A 0.25 coefficient means nothing to most readers.

-Problem 2: P-value Presentation Inconsistent

GDP per capita for males shows p=0.192 (non-significant) yet is retained in final model. This contradicts the stated backward elimination approach. Recommendation: Either remove non-significant variables or explain: “GDP per capita was retained despite p>0.05 due to theoretical importance and to maintain model comparability across sexes.”

-Problem 3: Direction of Effects Not Always Intuitive

Several findings require explanation: Why would alcohol use be negatively associated with female self-harm (Table 2)? Why would rule of law be positively associated with self-harm for both sexes? Why would labor force participation increase female self-harm?

Recommendation:

Add a results paragraph specifically addressing counterintuitive findings: “Several associations warrant careful interpretation. Rule of law showed positive associations, potentially reflecting better detection and reporting in well-governed countries rather than causal harm. Similarly, the negative association between alcohol use and female self-harm may reflect cultural factors, with countries having higher female alcohol use also having more gender-egalitarian norms that are protective for other reasons.”

-Problem 4: Cross-Sex Comparisons Not Systematically Presented

The manuscript presents male and female models separately but doesn’t formally test whether associations differ significantly by sex.

Recommendation: Add interaction analysis.

-Problem 5: Temporal Dynamics Ignored

Fixed effects for year are included but not discussed. Are there secular trends independent of measured contextual factors?

Recommendation:

Report: “After accounting for contextual factors, significant year fixed effects remained (F-test p<0.001), indicating unmeasured temporal trends such as changing diagnostic practices, evolving social media influence, or global policy shifts affecting self-harm incidence.”

5. DISCUSSION

5.1 Main Findings (Lines 92-100)

-Problem 1: Redundant with Results

The first paragraph largely repeats Results without interpretation.

-Problem 2: Sex Differences Understated

Lines 98-100 briefly mention sex differences but don’t emphasize this as a major finding.

5.2 Comparison with Existing Literature (Lines 102-139)

-Problem 1: Insufficient Critical Engagement

The discussion largely confirms previous findings without critical analysis of discrepancies or contradictions.

-Problem 2: Mechanistic Explanations Superficial

Lines 110-113 on substance use mention “pathways” but don’t elaborate sufficiently.

-Problem 3: Governance Findings Insufficiently Explored

Lines 114-118 on rule of law and corruption are underdeveloped given their theoretical importance.

-Problem 4: Adolescent Fertility Discussion Lacks Depth

Lines 119-124 are superficial given the strong associations found.

Recommendation:

Elaborate with policy implications: "Adolescent fertility’s strong association with self-harm, particularly among females, implicates multiple mechanisms: (1) STIGMA AND SOCIAL EXCLUSION: teenage pregnancy often results in school expulsion, family rejection, and community ostracism in many LLMICs, (2) ECONOMIC VULNERABILITY: early childbearing limits educational and economic opportunities, perpetuating poverty, (3) RELATIONSHIP VIOLENCE: adolescent pregnancies often occur in contexts of intimate partner violence or coercion, (4) MATERNAL MENTAL HEALTH: postpartum depression and anxiety are elevated among adolescent mothers with limited support. Critically, this suggests integrated reproductive health and mental health services for adolescents could yield suicide prevention benefits. Programs providing educational continuity, economic support, and mental health screening for pregnant/parenting adolescents warrant evaluation as self-harm prevention strategies. The association among males, though weaker, may reflect community-level effects or male partners' distress."

-Problem 5: Labor Force Participation Findings Require Gender Analysis

Lines 129-132 mention this contradictory finding but explanation is weak.

-Problem 6: HIV Discussion Too Brief

Lines 133-135: Given the finding’s importance, especially for males, this deserves more attention.

-Problem 7: Macro-Level Indicators Discussion Lacks Policy Implications

Lines 136-139 on SDI and GDP are descriptive without policy guidance.

5.3 Strengths and Limitations

-Problem: Limitations Section Too Brief and Uncritical

Lines 147-157 acknowledge some limitations but several critical ones are missing or understated.

6. Strengths and Limitations

While the study demonstrates strengths in geographic coverage and sex-specific analyses, several critical limitations are inadequately addressed or entirely absent. The reliance on GBD estimates with varying data quality across LMICs, particularly those dependent on sparse data and extensive modeling, is acknowledged but not sufficiently interrogated through sensitivity analyses or quality stratification. The ecological fallacy problem is underemphasized—national-level associations cannot inform individual-level risk and may reflect unmeasured confounding rather than causal relationships. Reverse causality remains unaddressed despite the cross-sectional design. Most concerning is the extensive unmeasured confounding: mental health service accessibility, suicide prevention policies, cultural attitudes toward suicide, means restriction (pesticides, firearms), and within-country heterogeneity are all absent, yet the low R² values suggest these unmeasured factors likely explain far more variance than measured predictors. Measurement heterogeneity across contextual indicators and selection bias from excluding conflict-affected nations further limit interpretation. The absence of comprehensive sensitivity analyses and multiple testing corrections undermines confidence in result robustness.

7. Implications and Future Directions

The implications section lists policy recommendations but lacks critical evaluation of evidence strength and feasibility given the study’s limitations. How can the authors justify specific policy actions when ecological associations preclude causal inference and unmeasured confounding is pervasive? The research recommendations are generic rather than specifically addressing the study’s methodological gaps. What concrete steps are proposed to overcome data quality issues, establish temporal precedence, or address the ecological fallacy? The discussion should acknowledge that findings generate hypotheses requiring validation through individual-level longitudinal studies rather than directly informing policy. The authors must clarify what can and cannot be concluded from this ecological analysis and provide a more realistic roadmap for bridging current evidence gaps.

8. REFERENCES

-Problem 1: Insufficient Recent Literature (2024-2025).

-Problem 2: Reference Formatting Inconsistencies

Cambridge Prisms: Global Mental Health uses a specific reference style (appears to be modified Harvard). Review their author guidelines carefully. Issues identified:

Journal abbreviations inconsistent: Some references use full journal names (“Journal of Affective Disorders”), others use abbreviations. Standardize according to journal requirements.

DOI formatting: There are multiple formatting errors in the DOIs of the references section, such as: “https://doi.org/https://doi.org/......”.

Issue numbers: Some references include issue numbers in parentheses, others don’t. Verify journal requirements.

-Problem 3: Key Missing References

Given the topic, notable absences include:

WHO technical documents on suicide prevention (recent updates);

Recent Lancet Commission on adolescent health and wellbeing;

Key papers on social determinants of mental health in LMICs (beyond Lund et al. 2018);

Recent papers on gender and suicide in developing countries;

Methodological papers on ecological study designs and their limitations;

Papers on data quality in GBD estimates for LMICs.

9. TABLES AND FIGURES

Table 1 (Males) and Table 2 (Females):

-Problem 1: Table Titles Lack Key Information

Current titles are generic. Titles should be self-explanatory.

Recommendation:

Revise to: “Table 1: Fixed-effects regression model of associations between country-level contextual factors and self-harm incidence rates among male adolescents (ages 10-19) in 77 low- and lower-middle-income countries, 2000-2021”

-Problem 2: Non-Significant Results Included

GDP per capita (males, p=0.192) is non-significant but included. Either remove or add footnote explaining retention rationale. The p-value of 0.000 should be changed to <0.001.

Figure 1 (AAPC Maps):

-Problem 1: Legend Clarity

Ensure legend clearly indicates: What AAPC values are statistically significant. Whether non-significant trends are shown (and how). Sample size (n=77 countries)

10. OTHER

-The overall presentation of the manuscript needs further refinement and revision, as there are some grammatical or tense errors, unidiomatic English expressions, etc. For example, The “METHODS” section mixes tenses. “The study utilizes data” should be “The study utilized data” (tense consistency - the methods section should be in the past tense).

-First use in title as “Low- and Lower-Middle Income Countries” - should define abbreviation in abstract first use: “low- and lower-middle-income countries (LLMICs)”

-Some acronyms and their full names appear repeatedly in the main text, such as GBD and its full name. All abbreviations defined at first use in each major section (Abstract, Main Text).

-Critical Error: Line 68 defines VIF as “variable impact factor” - this is INCORRECT. VIF stands for “variance inflation factor,” a measure of multicollinearity in regression models. This must be corrected.

11. FINAL RECOMMENDATION: MAJOR REVISION

This manuscript addresses an important and understudied topic—adolescent self-harm in low- and lower-middle-income countries—and provides valuable insights into sex-specific contextual determinants. The large-scale analysis covering 77 countries over 22 years represents a substantial contribution to the global mental health literature.

However, significant revisions are required before the manuscript can be considered for publication:

Essential Revisions:

-Strengthen theoretical framework in Introduction with clear mechanistic pathways;

-Add 2024-2025 literature (minimum 5-8 recent high-quality papers);

-Expand Discussion of counterintuitive findings (rule of law, alcohol use, labor force participation);

-Address data quality concerns more thoroughly, including sensitivity analyses restricted to high-quality data countries;

-Correct critical error: VIF definition (line 68);

-Expand limitations section comprehensively addressing all issues raised;

-Conduct and report sensitivity analyses (conflict countries excluded, lagged exposures, regional stratification, data quality tiers);

-Improve interpretive clarity of statistical findings with practical effect size examples;

-Verify and complete all reference page numbers and formatting per journal requirements;

-Address all grammatical and stylistic issues identified throughout.

Recommended Revisions:

-Add forest plots or scatter plots showing key associations;

-Provide more detailed policy recommendations with prioritization;

-Expand discussion of mechanisms linking contextual factors to self-harm;

-Include data quality metrics in supplementary materials;

-Clarify self-harm definition and GBD limitations more explicitly.

Minor Revisions:

-Improve table footnotes with fuller explanations;

-Enhance figure captions;

-Ensure consistent abbreviation usage;

-Improve abstract with background statement;

-Revise Impact Statement for greater clarity and impact.

# Reviewer report: Review: The trend of self-harm incidence rates among adolescents in low- and lower-middle-income countries and its associated contextual factors — R0/PR4

**DOI:** 10.1017/gmh.2026.10241.pr4

**Authors:** Prodyut Roy

**Affiliations:** Educational Psychology, Independent Scholar, Bangladesh

Overall statement:

I have not found a significant impact of this manuscript, and it lacks coherence. This manuscript mismatches the terms related to the study of suicidal behaviour and self-harm. This manuscript is not ready for publication. The authors need to spend significant time to improve the quality of this manuscript.

Abstract: The title proposes to examine the trend of self-harm incidence among adolescents, but the first sentence of the abstract does not convey the same meaning as the title suggests. The trend of self-harm incidence and incidence rates are not the same. Hope the authors will address this issue. Could you please use the full meaning instead of acronyms (e.g., LLMICs) when it is applied for the first time?

Impact Statement

Please revamp the impact statement, focusing on the impact of this study, not writing an abstract.

Introduction

For line 6 (Page 4), please delete ‘estimates’ or ‘according to’ to make the statement meaningful. For lines 10-13 (Page 4), the statement conveys several contextual meanings; for example, (1) self-harm is a risk factor for suicide, (2) self-harm increases the risk of suicide by more than 30 times compared to the general population. For this statement, I have the following observations:

1. I have gone through the cited references and found that three cited articles do not indicate the same findings because their research areas were different. For instance, Aggarwal et al. (2017) conducted a systematic review of protective and risk factors for self-harm; hence, they did not claim any issues the authors shared in this statement.

2. Self-harm has its types based on the intention or non-intention of suicidal behaviour. In your study, please articulate the type of self-harm you are going to examine.

3. Please note that suicide has its distinct meaning in the study of suicidal behaviour. Suicide and suicidal behaviour are not the same. I understood that you used them as synonymous. If yes, I request you to make them distinct; otherwise, it will confuse the readers and cause chaos in the literature.

4. For lines 10-13, I request you to cite the context independently. For example, ‘given that self-harm is a well-known risk factor for suicide (cite, year) and increases the risk of suicide by more than ……..(cite, year).

For lines 20-21 (page 4), please claim your statement with a recent study because suicide is the third leading cause of death globally, and you are claiming this statement with a reference which was conducted in 1989.

For lines 21-35 (page 4), at first, you stated that the self-harm rate was reduced globally from 1990 and 2021. Could you claim this factual information with a reference? Later, you mentioned that these reduction rates were not observed in Central and Tropical Latin America and Southern sub-Saharan Africa. My question is, who observed it? Please make it credible with proper citations. Please include citations for the claim that many LLMICs are located in those areas. After “in particular” you mentioned previous studies, but you did not clearly indicate which studies, because you have cited only one article (Yan et al. 2024) for this, but you mentioned studies (plural). For line 31, it is not clear which burden you are talking about. I did not grasp the highlighting point you want to illustrate, because this paragraph portrays several contexts and lacks the coherence which misguides the readers. I request you to break down this big paragraph into two or more paragraphs with coherence.

For lines 38-39 (page 4), you mentioned ‘studies’, but you did not cite them. Could you please claim your statement with citations, which will act as evidence of how suicidal behaviour and self-harm have been linked to narrated factors?

For lines 41-45 (page 4), please be specific in citing; for example, all cited references do not explore all factors together. Your cited style indicates that all the findings of your cited references are the same, which is not correct.

For lines 46-48 (page 4), the authors suddenly introduce a conceptual framework; they did not explain the conceptual framework or how it is related to this study. However, as a reader, I am perplexed by the term “contextual factors”. Could you please define what factor can be contextual factor is?

For lines 58-60 (page 4), as a peer reviewer and reader of mental health-related articles, I can state that you will find enough published research on suicidal behaviour and self-harm behaviour of adolescents in LMICs. I request you to establish your claim against scarcity in research on suicidal behaviour and self-harm behaviour of adolescents in LMICs with a citation.

For lines 11-16 (page 5), the word ‘establishing’ does not convey a precise and concise meaning for research objectives. Please find an appropriate term to express the research objectives. In addition, I request you cite for age range for the adolescent group of people, because 19 could fall under adulthood for some countries, particularly for LLMICs.

Before the last paragraph of the introduction section, is it possible to include one or two paragraphs explaining the significance and novelty of this study? I would appreciate it if you could describe the theoretical framework which supports your findings. Please also illustrate how this study will address the gap you have already explained in earlier paragraphs.

Methodology

I request you to revamp the methodology sections addressing the following concerns:

1. Please justify why you have employed an ecological study design to address the objectives of this study.

2. I understand you have utilised publicly available secondary sources of data. As researchers, it is your responsibility to ensure that the secondary sources of data have been collected following the ethical standards. The readers of this manuscript are curious to know how the researchers of the primary sources of data practised the ethical considerations.

3. The methodology section is not the ideal section to define any terms if it is not related to your methodology; hence, the definition of self-harm, contextual factors and other relevant terms should be introduced in the introduction section.

4. You have provided some links to access the data and relevant information to support your statement; it is better to follow the rules of in-text citation.

Now, I am offering you the line-by-line feedback to improve the quality of your manuscript. For lines 24-26 (page 5), please cite against Global Burden of Disease study.

For lines 26-29 (page 5), please use the past tense form for ‘includes’ and ‘utilizes’. For line 46 (page 5), please cite the World Bank data related to the categorisation of LLMICs.

For line 48 (page 5), restructure the in-text citation format; for example, “Utilizing ….factors by Lange et at. (2023) and Rajkumar (2023), we….”

For line 54 (page 5), please write the full abbreviation for SDI. For contextual factors, you have included several factors, and you declared that you have selected these factors from previous literature. My question: Why have you not selected all factors and reported them in the result section, though you have included several factors?

For line 11 (page 11), covariates and associated factors are not the same; readers can confuse the term ‘covariate’. Could you please explain how you have established the contextual factors as sex-specific, which were displayed in Supplementary Table 1? Moreover, please clearly articulate whether these factors serve as the factors for suicide or/and self-harm.

Data analysis

Please use the past tense form and cite your justification. For line 4 (page 7), for your information, I have checked serval times but did not find supplementary tables 4 and 5.

For line 51 (page 7), for the identification of contextual factors, you have conducted a series of regression analyses, but in lines 49-50 (page 5), you have stated that you have utilised previous literature to obtain contextual factors. Could you please be precise and concise regarding the process of the identification of contextual factors?

Results

Please be consistent with the objectives of this study; ‘self-harm incidence’ and ‘self-harm incidence rate’ do not convey the same meaning. For example, the title of Figure 1 illustrates the self-harm incidence, not the incidence rate.

For trend analysis, I understand the objectives of your study; sex-specific analysis will help to comprehend the difference between trends for male and female adolescents‘ self-harm incidence rate. I do not know whether it is possible or not to make a comparison between male and female adolescents’ self-harm incidence rates on a country basis. Is there any country where the self-harm incidence rate, for example, for males decreased but for females increased, or vice versa?

For contextual factors

Before displaying the contextual factors, it would be ideal practice if you declare the final list of contextual factors identified from this study. Why will these factors be contextual factors? Please establish it with proper citations. The result section is not ideal for the sections for identification and declaration of the final factors. Please put them in the appropriate sections.

For lines 50-53 (page 7), you are explaining factors for males, but referring to women’s ages (in line 53).

Discussion

Before going to line-by-line comments, I have some observations:

• Self-harm falls under the school of psychology; it is established that bio-psycho-social factors are associated factors for self-harm incidence. Could you please justify the reason for omitting psychological and socio-cultural factors for self-harm incidences?

• Please delete the main findings section; the results section is the appropriate section to describe the main findings.

• There is no need to put a sub-title ‘comparison with existing literature’, because comparison with existing literature is one of the core components for discussion sections to support and discuss your findings.

For lines 9-10 (page 9), could you please include or cite against previous studies? For lines 10-13, you did not include the difference between male and female adolescents’ self-harm incidence rate in the results section. Please include the difference first in the result sections; afterwards, explain here in the discussion section. Please also illustrate the possible explanation of the gradually increasing and decreasing self-harm incidence rates for sex-specific populations.

For lines 14-28, I did not comprehend the relevance of these statements. Could you please revamp them or justify their relevance for the discussion section?

For lines 30-33, you have suddenly introduced a new term, ‘cross-cutting’ contextual factors, cross-cutting contextual factors, and contextual factors are not the same. Please explain first how drug use, adolescent fertility rate, and other mentioned factors are cross-cutting contextual factors.

You wrote ‘previous literature supports..”, we do not know which literature supports your claim. Could you please cite it? For lines 33-40, all of your cited evidence is on suicidal behaviour, and your research on self-harm. Please note that suicidal behaviour and self-harm have distinct differences; hence, research on suicidal behaviour would not justify the explanation for the findings of research on self-harm.

For lines 46-48, a lower suicide rate in the countries of lower levels of corruption does not mean that lower levels of corruption can be a causal or associated factor for suicidal behaviour. Please correct me if I am wrong. Again, how can findings from research on suicide be appropriate for the research on self-harm? For your reference, suicidal behaviour includes suicidal ideation/thoughts, suicidal attempt, and suicide; hence, suicide and self-harm are not the same. Self-harm with the intention of suicide can be a factor for suicidal behaviour (e.g., suicidal attempt and suicide).

For lines 50-53, please establish coherence between statements.

For lines 55-56, please claim your assumption through proper citations. For lines 57-60, please be informed that adolescent pregnancy and pregnancy out of marriage are not the same. Please justify how pregnancy can be a risk factor for suicide.

For lines 4-6 ( page 10), please justify with proper citations how adolescent pregnancy is seen as taboo and how it is associated with self-harm behaviour.

I request that the authors revamp the discussion sections. Based on the given feedback, please improve the depth of the discussion section.

# Editor report: Recommendation: The trend of self-harm incidence rates among adolescents in low- and lower-middle-income countries and its associated contextual factors — R0/PR5

**DOI:** 10.1017/gmh.2026.10241.pr5

**Affiliations:** Mount Sinai School of Medicine, United States

Please addess al concerns from reviewers. Consider inclusion of most recent GBD estimates. Play careful attention to methodological flaws identified in the reviewers‘ reports and address them. We cannot ensure acceptance of a revised version, but the paper cannot be considered as is. We are happy to review a new version and will pay attention to your responses to reviewers’ comments.

# Editor report: Decision: The trend of self-harm incidence rates among adolescents in low- and lower-middle-income countries and its associated contextual factors — R0/PR6

**DOI:** 10.1017/gmh.2026.10241.pr6

**Affiliations:** Social Work, University of South Florida, United States

No accompanying comment.

# Reviewer report: Review: The trend of self-harm incidence rates among adolescents in low- and lower-middle-income countries and its associated contextual factors — R1/PR8

**DOI:** 10.1017/gmh.2026.10241.pr8

May I request a document that is fresh from proofreading, I am having a hard time on understanding the whole document due to incomplete sentences that has to be jumped from one point to another. Thank you!

# Reviewer report: Review: The trend of self-harm incidence rates among adolescents in low- and lower-middle-income countries and its associated contextual factors — R1/PR9

**DOI:** 10.1017/gmh.2026.10241.pr9

**Authors:** Prodyut Roy

**Affiliations:** Educational Psychology, Independent Scholar, Bangladesh

Dear Authors,

Thank you for carefully addressing all the reviewers’ comments and revising the manuscript accordingly. The revisions have substantially improved the quality of the paper, and the manuscript now meets the required scholarly standards.

I am pleased to accept the manuscript for publication, with one minor suggestion. I encourage you to further improve the coherence and flow of the Discussion section, ensuring that the key arguments are more clearly connected and that the section offers a stronger, more engaging synthesis of the findings. Giving the Discussion a clearer narrative structure will enhance its overall impact.

Thank you for your valuable contribution, and I wish you every success with the final version of the manuscript.

# Reviewer report: Review: The trend of self-harm incidence rates among adolescents in low- and lower-middle-income countries and its associated contextual factors — R1/PR10

**DOI:** 10.1017/gmh.2026.10241.pr10

Reviewer Comments for Manuscript GMH-2025-0295

Title: The Trend of Self-Harm Incidence Among Adolescents in Low- and Lower-Middle Income Countries and Its Associated Contextual Factors

General Comments

The current manuscript, which examines the trends in adolescent self-harm incidence across 77 low- and lower-middle income countries (LLMICs) from 2000 to 2021 and investigates the associated contextual factors using data from the Global Burden of Disease 2021 database. The study addresses an important and underexplored area in global mental health research, particularly given that the majority of global suicides occur in low- and middle-income settings.

The manuscript has several strengths. The topic is highly relevant and timely, addressing a significant gap in the literature regarding adolescent self-harm in resource-limited settings. The use of joinpoint regression analysis to examine temporal trends across multiple countries is methodologically appropriate, and the sex-stratified analytical approach is commendable. The study utilizes a large dataset spanning 22 years and 77 countries, which enhances the generalizability of the findings. Additionally, the inclusion of multiple contextual factors from reputable data sources (World Bank, UNDP, GBD) strengthens the analytical framework.

However, there are several methodological and presentation issues that require attention before this manuscript can be considered for publication. My detailed comments and suggestions are outlined below.

Specific Comments

1. Abstract – Abbreviations

The abbreviation “LLMICs” appears in the abstract without its full form being provided at first mention. I recommend spelling out “Low- and Lower-Middle Income Countries (LLMICs)” upon its initial appearance in the abstract to ensure clarity for readers.

2. Missing References for Evidence-Based Statements

Several statements in the manuscript present empirical claims without supporting references. For example, the statement “However, recent trends indicate a global decline in adolescent suicide and self-harm. Between 1990 and 2021, the suicide mortality rate among young adults and adolescents decreased at an annual percentage change rate of -1.6%” requires appropriate citations. The authors should carefully review the manuscript and ensure that all evidence-based assertions are adequately supported by references.

3. Clarity of Writing

Some passages in the manuscript lack clarity and may impede reader comprehension. For instance, the sentence in the abstract—“Average annual percentage changes were negative in 40 countries for males and 47 for females but positive up to 2.16%, and 3.64%, respectively, elsewhere”—presents several issues: (a) the referent of “respectively” is ambiguous, requiring readers to infer that 2.16% corresponds to males and 3.64% to females; (b) the information density is excessive, as one sentence attempts to convey two sexes, two trend directions, different country counts, and specific numerical values simultaneously; (c) the presentation is asymmetric, with negative changes reported only as country counts while positive changes are reported only as percentage caps; and (d) “elsewhere” is vague regarding the number of countries involved. I recommend that the authors revise such sentences for improved clarity and readability.

4. Redundant Presentation of Abbreviations and Full Terms

There are instances where abbreviations and their full forms are presented redundantly. For example, “global burden of disease (GBD)” appears to be defined more than once. The authors should conduct a thorough review to ensure that each abbreviation is defined only at first mention and used consistently thereafter.

5. Justification for Analytical Decisions in Data Analysis

In the data analysis section, the rationale for model selection, the threshold for high correlations (i.e., r = ±0.7 to ±1.0), and the criteria for variable retention require clearer justification. The authors should provide methodological references supporting these analytical decisions.

6. Joinpoint Regression Analysis – Methodological Justification

Joinpoint regression analysis appears to be a central analytical method in this study. The authors should elaborate on the methodological advantages of this approach and describe how the accuracy of the identified joinpoints was verified or validated.

7. Hausman Test – Criteria for Model Selection

The manuscript states that the Hausman test was employed to determine whether fixed-effects or random-effects analysis was appropriate. The authors should clarify the specific criteria and decision rules used in interpreting the Hausman test results.

8. Age Discrepancy in Contextual Factors

The study focuses on adolescents aged 10-19 years; however, the “Contextual factors for self-harm incidence” section includes variables pertaining to the 15-24 age group, such as “young people (ages 15-24) newly infected with human immunodeficiency virus.” The authors should explain the rationale for including data from this broader age range and discuss whether and how this age mismatch may influence the results or their interpretation.

9. Rationale for Inclusion of Contextual Factors

The methods section indicates that numerous contextual factors were considered in the analysis. The authors should provide a more explicit justification for the selection and inclusion of these specific factors, ideally grounded in theoretical frameworks or prior empirical evidence.

10. Insufficient Discussion of Key Findings – Alcohol Use Among Females

The results indicate that among females, higher alcohol use was negatively associated with self-harm incidence rates. While most studies report positive associations between alcohol use and self-harm in males, findings in females are more heterogeneous, with some studies reporting no significant association. This finding in the present study appears noteworthy and potentially counterintuitive, yet the discussion section does not provide an in-depth exploration of this result. I recommend that the authors offer a more substantive discussion of this finding rather than simply listing evidence or restating the results.

11. Outdated References

Some references cited in the manuscript are considerably dated. For instance, the citation supporting the claim that “suicide is the third leading cause of death among young people and self-harm often begins in adolescence” dates from 1989. Furthermore, the majority of references predate 2025. I recommend that the authors update the literature review to include more recent publications that reflect current trends and evidence.

12. Lack of Sex-Specific Policy Recommendations in the Conclusion

The results demonstrate that different contextual factors differentially affect self-harm incidence among male and female adolescents. However, the conclusion does not offer concrete, sex-specific policy recommendations. Given that the study design and analyses were stratified by sex, I would expect to see more nuanced, gender-informed policy implications derived from the findings. The current concluding recommendations appear overly general. The authors should consider providing actionable and specific policy suggestions that address the observed sex differences.

13. Apparent Contradiction Between Results and Conclusion Regarding Alcohol Use

The results suggest that alcohol use is negatively associated with self-harm incidence among females, which could paradoxically imply a protective effect. However, the conclusion states that “there is a need to address drug and alcohol use at the national level.” Given this intriguing finding, how such measures should be implemented becomes particularly important. Yet, I did not find any discussion addressing this apparent discrepancy or providing guidance on how to reconcile this result with policy recommendations.

14. Discussion of Joinpoint Regression Findings

The authors should consider adding a discussion of the potential reasons underlying the key joinpoints (inflection points) identified through the joinpoint regression analysis. Such discussion would enhance the interpretive value of the trend analysis.

15. Limitations Section – Data Quality and Sensitivity Analysis

Given that the analysis is restricted to LLMICs, the authors should acknowledge potential issues related to data collection in these settings, including possible underreporting or data gaps attributable to economic and developmental constraints. Furthermore, the authors note that countries and variables with substantial missing data were excluded, and multiple imputation was performed using data from neighboring countries within the same region and income classification. It would be advisable to conduct sensitivity analyses to verify the robustness of the results. Additionally, a methodological discussion of how these data handling procedures may have influenced the findings is warranted to ensure transparency regarding the reliability and validity of the results.

16. Detailed Reporting of Missing Data Handling Procedures

The methods and data analysis sections should provide more detailed descriptions of the criteria, methods, and operational procedures used for excluding missing data and for conducting multiple imputation. Enhanced transparency in this regard would improve the reproducibility of the study.

# Editor report: Recommendation: The trend of self-harm incidence rates among adolescents in low- and lower-middle-income countries and its associated contextual factors — R1/PR11

**DOI:** 10.1017/gmh.2026.10241.pr11

**Affiliations:** Mount Sinai School of Medicine, United States

Thanks for addressing most reviewers' comments. Please pay attention to some concerns highlighted by one of the reviewers. In addition, see below my own overview of what is most relevant to address

The manuscript addresses an important and underexamined topic—adolescent self-harm trends across 77 low- and lower-middle income countries using GBD data—and has several strengths, including its large scope and sex-stratified analyses. However, further revision is required before it can be considered for publication. In particular, the manuscript needs clearer methodological justification. The rationale for key analytical decisions (e.g., model selection procedures, correlation thresholds, interpretation of the Hausman test, and joinpoint specification) should be more transparently explained and appropriately referenced to ensure reproducibility and interpretability.

There are also important interpretive issues that require clarification. The inclusion of contextual variables drawn from the 15–24 age group in a study focused on ages 10–19 should be explicitly justified and discussed. In addition, the negative association between alcohol use and female self-harm incidence is insufficiently examined and appears inconsistent with the policy recommendations in the conclusion. This finding warrants deeper discussion and clearer reconciliation with the proposed implications.

Finally, the manuscript would benefit from a more explicit discussion of data limitations, including missing data handling and imputation procedures, particularly given the reliance on national-level estimates in resource-constrained settings. Strengthening these methodological and interpretive elements would substantially improve the clarity and credibility of the study. I therefore recommend revision prior to further consideration.

# Editor report: Decision: The trend of self-harm incidence rates among adolescents in low- and lower-middle-income countries and its associated contextual factors — R1/PR12

**DOI:** 10.1017/gmh.2026.10241.pr12

**Affiliations:** Social Work, University of South Florida, United States

No accompanying comment.

# Reviewer report: Review: The trend of self-harm incidence rates among adolescents in low- and lower-middle-income countries and its associated contextual factors — R2/PR14

**DOI:** 10.1017/gmh.2026.10241.pr14

References across the article is a lot, for example in line 43, the referred article or source was noted at 29, however, looking at the references there are only 14 references that were placed.

I’m not sure if it was just forgotten or if the paper could be written by a hallucinating generative AI. I hope it’s the former, please revise the paper, complete the references.

This is important, as there had been multiple cases of paper where references were non existent and it was generated by LLMs, which resulted to it being redacted.

This was randomly checked by me, so please recreate the references and add numbers to it so we can cross examine the references. Thank you so much!

# Reviewer report: Review: The trend of self-harm incidence rates among adolescents in low- and lower-middle-income countries and its associated contextual factors — R2/PR15

**DOI:** 10.1017/gmh.2026.10241.pr15

Dear Authors,

Thank you for your careful and thorough revisions in response to the reviewer comments. These modifications have notably improved the overall quality of the manuscript. At this stage, I am satisfied that the manuscript meets the required academic standards.

I am pleased to recommend this manuscript for acceptance and publication. However, I would like to offer one minor suggestion: I encourage the authors to consider incorporating additional up-to-date references, particularly high-quality and relevant works published in 2025 and 2026. The inclusion of such recent literature would further strengthen the currency and cutting-edge nature of this research, enhancing its overall scholarly impact.

# Editor report: Recommendation: The trend of self-harm incidence rates among adolescents in low- and lower-middle-income countries and its associated contextual factors — R2/PR16

**DOI:** 10.1017/gmh.2026.10241.pr16

**Affiliations:** Mount Sinai School of Medicine, United States

Thank you for addressing the reviewers‘ and editors’ comments.

# Editor report: Decision: The trend of self-harm incidence rates among adolescents in low- and lower-middle-income countries and its associated contextual factors — R2/PR17

**DOI:** 10.1017/gmh.2026.10241.pr17

**Affiliations:** Social Work, University of South Florida, United States

No accompanying comment.

# Editor report: Recommendation: The trend of self-harm incidence rates among adolescents in low- and lower-middle-income countries and its associated contextual factors — R3/PR19

**DOI:** 10.1017/gmh.2026.10241.pr19

**Affiliations:** Mount Sinai School of Medicine, United States

No accompanying comment.

# Editor report: Decision: The trend of self-harm incidence rates among adolescents in low- and lower-middle-income countries and its associated contextual factors — R3/PR20

**DOI:** 10.1017/gmh.2026.10241.pr20

**Affiliations:** Social Work, University of South Florida, United States

No accompanying comment.
